# Relaxed purifying selection in autopolyploids drives transposable element over-accumulation which provides variants for local adaptation

**DOI:** 10.1038/s41467-019-13730-0

**Published:** 2019-12-20

**Authors:** Pierre Baduel, Leandro Quadrana, Ben Hunter, Kirsten Bomblies, Vincent Colot

**Affiliations:** 1 Institut de Biologie de l’Ecole Normale Supérieure (IBENS), Centre National de la Recherche Scientifique (CNRS), Institut National de la Santé et de la Recherche Médicale (INSERM), Ecole Normale Supérieure, PSL Research University, 75005 Paris, France; 2 Department of Organismic and Evolutionary Biology, Harvard University, Cambridge, MA 02138 USA; 3 ETH Zürich, 8092 Zürich, Switzerland

**Keywords:** Evolutionary genetics, Mobile elements, Polyploidy in plants, Plant evolution

## Abstract

Polyploidization is frequently associated with increased transposable element (TE) content. However, what drives TE dynamics following whole genome duplication (WGD) and the evolutionary implications remain unclear. Here, we leverage whole-genome resequencing data available for ~300 individuals of *Arabidopsis arenosa*, a well characterized natural diploid-autotetraploid plant species, to address these questions. Based on 43,176 TE insertions we detect in these genomes, we demonstrate that relaxed purifying selection rather than transposition bursts is the main driver of TE over-accumulation after WGD. Furthermore, the increased pool of TE insertions in tetraploids is especially enriched within or near environmentally responsive genes. Notably, we show that the major flowering-time repressor gene *FLC* is disrupted by a TE insertion specifically in the rapid-cycling tetraploid lineage that colonized mainland railways. Together, our findings indicate that tetrasomy leads to an enhanced accumulation of genic TE insertions, some of which likely contribute to local adaptation.

## Abstract (web summary)

Why transposable elements (TEs) accumulate in polyploids and the evolutionary implications remain unclear. Here, the authors show that following whole genome duplication, relaxed purifying selection is the main driver of TE over-accumulation, which provides variants for rapid local adaptation.

## Introduction

Eukaryotic genomes display remarkable variation in ploidy as well as in size. This is particularly true of plant genomes, the evolution of which has been punctuated by numerous whole genome duplication (WGD) events^1–3^. Moreover, polyploidization is often associated with an increase in transposable element (TE) content, which further exacerbates the inflation of genome size^3,4^. This association has long been seen as supporting the “genome-shock” hypothesis initially proposed by Barbara McClintock^5^, where genomic instabilities associated with genome doubling could trigger transposition bursts. However, the possibility of such bursts has been studied primarily in allopolyploids, where the effects of WGD and hybridization are confounded (e.g. refs. ^6,7^). Furthermore, the doubling of genome copies should double the mutation rate per individual and reduce the selective pressure exerted on recessive deleterious mutations^8^, especially in autopolyploids where all homologous chromosomes segregate randomly (polysomic masking). Therefore, the loss-of-function mutations typically caused by TE insertions could readily over-accumulate in polyploids, even in the absence of transposition bursts.

These two non-mutually exclusive scenarios to account for the increased TE content of polyploids are still presented indiscriminately (e.g. ref. ^9^) because of a lack of experimental support for one or the other^10,11^. Yet, their evolutionary implications differ substantially: while transposition bursts resulting from the WGD event are expected to impact the fitness of neo-polyploids in the first few generations after they are formed^12,13^, the effects of relaxed purifying selection due to polysomic masking would only be perceived many generations downstream, as a result of the progressive accumulation of new TE insertions. In any case, the slower allele dynamics caused by relaxed purifying selection in polyploids^14^ do not necessarily imply slower rates of adaptation. Indeed, when environments change, previously deleterious alleles can turn beneficial. Thus, the increased amount of standing variation in polyploids is expected to result in faster adaptive responses^15^. Moreover, polyploids may also benefit directly from their higher mutation rate per individual, if some mutations are at least partially dominant^15^.

Here, we set out to determine the respective importance of the two possible routes to TE accumulation following WGD using *Arabidopsis arenosa* as a model system. This plant species occurs in both diploid and autotetraploid forms across Central and Northern Europe^16^. The autotetraploids are fully tetrasomic and originated in the Western Carpathians from a single recent WGD event^16^ (~60 kyr ago). They rapidly expanded and now occupy a broader ecological range than their diploid progenitors^17^. Notably, as frequently reported for polyploids^18,19^, one *A. arenosa* tetraploid lineage successfully invaded a novel ruderal habitat, where they adopted a weedy life-cycle not shared by diploids or non-ruderal tetraploid *A. arenosa*^20,21^. This notwithstanding, the largely overlapping ecological niches and the recent divergence between the two *A. arenosa* lineages allows for cross-ploidy genomic comparisons^22^.

Using the most comprehensive diploid-autopolyploid dataset to date^22^, we characterize TE dynamics across diploid and tetraploid *A. arenosa* genomes. First, we assess how the relaxation of purifying selection impacted TE dynamics in autotetraploids. Second, we investigate whether a transposition burst was associated with the WGD event that all *A. arenosa* tetraploids trace back to. Finally, we ask whether and how TE dynamics in autotetraploids might contribute to their adaptive potential.

## Results

### TE landscapes in diploid and tetraploid *A. arenosa* genomes

Genome resequencing data are available for 105 diploid and 181 tetraploid individuals of *A. arenosa*, corresponding to 15 and 23 distinct populations covering the entire range of the species, respectively^22^. In the absence of a reference *A. arenosa* genome sequence, we used that of the closely related *Arabidopsis lyrata* species^23^ as was done in previous SNP-based population genomic studies of *A. arenosa*^16,22,24^.

**Fig. 1:**
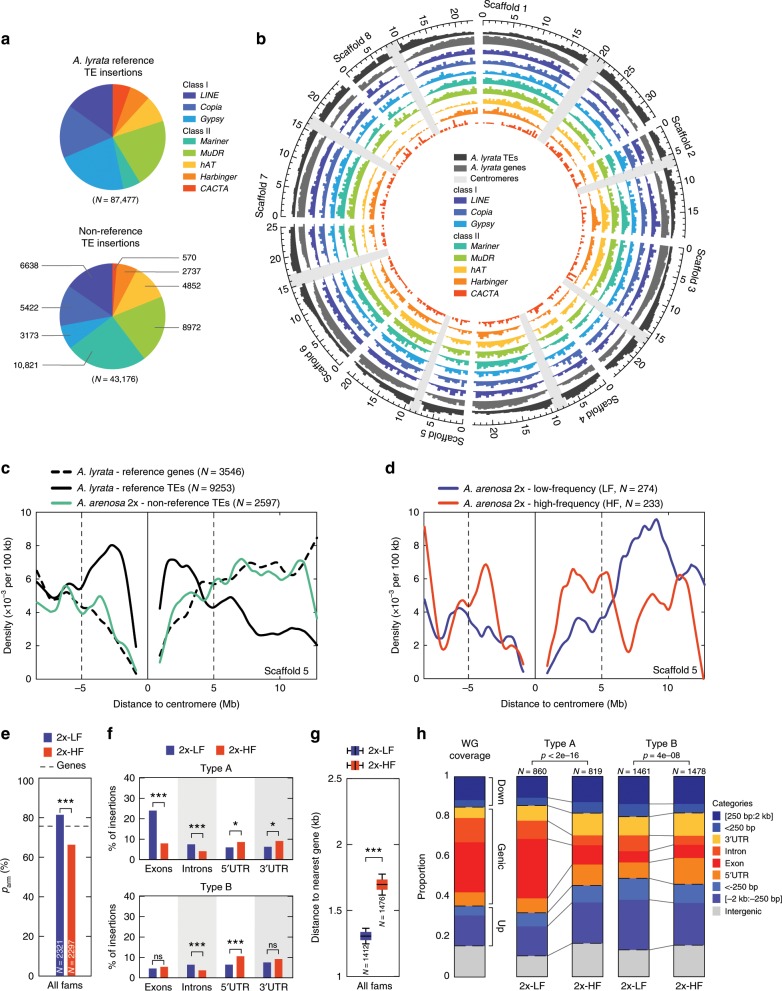
Natural selection shapes the TE landscape of A. *arenosa* diploids. **a** Distribution of reference TEs (upper chart) and non-reference (lower chart) TE insertions identified by SPLITREADER across the 8 class I & II TE superfamilies. **b** Chromosomal distributions of reference genes and TEs and of non-reference TE insertions by TE superfamily across the 8 scaffolds of the *A. lyrata* reference genome. **c** Density per 100 kb of reference genes and TEs and of non-reference diploid TE insertions across scaffold 5. **d** Density per 100 kb across scaffold 5 of low- and high-frequency TE insertions in diploids. **e** Fraction, *p*_arm_, within chromosome arms (>5 Mb away from centromeres) of low- and high-frequency TE insertions in diploids with *p*-values of *χ*^2^-test. (f*)* Fraction of low- and high-frequency TE insertions in diploids overlapping exons, introns, 5′ or 3′ UTRs for type A (upper panel, *n* = 860 2x-LF and *n* = 819 2x-HF) and type B (lower panel, *n* = 1461 2x-LF and *n* = 1478 2x-HF) superfamilies with *p*-values of *χ*^2^-test. **g** Boxplots of distance to nearest gene (kb) of low- and high-frequency non-genic TE insertions in diploids across 1000 bootstraps and *p*-value of *t*-test between ploidies. Boxplot center lines, median; box limits, upper and lower quartiles; whiskers, 9th and 91st quantiles. **h** Distribution of low- and high-frequency TE insertions in diploids across categories of insertions for type A and type B superfamilies compared to reference genome annotations with *p*-values of *χ*^*2*^-test. *p* *<* 0.001: ***; *p* < 0.01: **; *p* < 0.05: *; *p* ≥ 0.05: ns. The source data underlying Fig. 1b–h are provided as a Source Data file.

We assessed recent TE activity in *A. arenosa* using the SPLITREADER pipeline^25^ (see Methods), which enabled us to identify 43,176 non-reference TE insertions (hereafter simply designated as TE insertions) from split and discordant reads in the 286 re-sequenced *A. arenosa* genomes aligned on the *A. lyrata* reference genome. These TE insertions correspond to three class I (*LINE, Copia*, and *Gypsy* retrotransposons) and five class II (*Mariner, MuDR, hAT, Harbinger*, and *CACTA* DNA transposons) superfamilies, in proportions similar to those found in *A. lyrata*^26^ (Fig. 1a). However, unlike annotated TE sequences of the *A. lyrata* reference genome, which are heavily enriched towards pericentromeric regions, the TE insertions we detected are homogeneously distributed along chromosomes with no obvious pericentromeric bias (Fig. 1b).

### Purifying selection shapes TE landscapes in diploids

To characterize more precisely TE localization and dynamics we started with the 105 diploid (i.e. 2×) individuals. The density of TE insertions is higher on the chromosome arms than within 5 Mb of the centromeres and follows that of the genes rather than the TEs annotated in the *A. lyrata* reference genome (Fig. 1c). In order to test whether this higher density of TE insertions within chromosome arms reflects recent TE activity or detection biases against pericentromeric regions, we compared the distribution along chromosomes of those TEs at low- and high- carrier frequencies (2x-LF vs 2x-HF, see Methods). This parameter is a proxy for relative age when positive selection is rare, because recent TE insertions are unlikely to be shared by more individuals than older ones. Across all TE superfamilies, we observed a clear and consistent decrease (−13.5%, *χ*^2^-test *p* < 0.001) in the density and proportion (*p*_arm_) of HF compared to LF TE insertions within chromosome arms (Fig. 1d, e). Furthermore, outside of pericentromeric regions HF TE insertions were found significantly less frequently than LF TE insertions in gene-rich compared to gene-poor regions (*χ*^2^-test *p*-value < 1E−7, Table S1). This distribution shift was also associated with a deficit of HF TE insertions within genes, specifically within exons and introns (Fig. 1f), as well as near (<250 bp) genes (Fig. 1g). These observations are consistent with purifying selection acting to progressively filter out TE insertions from chromosome arms due to their generally deleterious effects on genes, as reported in *A. thaliana*^25^. In keeping with this interpretation, we found a significant association between changes in transcript levels and presence of TE insertions within or near (<250 bp) genes but not when further away (Supplementary Fig. 1a, b, see Methods). These effects, mostly negative, likely reflect the impact of TE insertions on the expression of nearby genes but could also be the result of insertion preferences for haplotypes already differentially expressed.

Further analysis revealed that the deficit of HF TE insertions within or near genes is most pronounced for the *Copia*, *Gypsy*, *CACTA*, and *hAT* TE superfamilies, which show an apparent insertion preference (evaluated using LF TE insertions) for genes, especially exons. Specifically, the proportion of these TE insertions that lie within exons and introns are much reduced (−20.0% and −4.2%, respectively) at high frequency (Fig. 1f), consistent with them having the most detrimental effects and therefore being the most rapidly purged by natural selection. TE insertions from these superfamilies are hereafter called “type A” (Fig. 1h and Supplementary Fig. 1c).

In contrast, HF as well as LF TE insertions from the other TE superfamilies (*LINE, Mariner, MuDR*, and *Harbinger*), thereafter called “type B”, are underrepresented within exons (Fig. 1f–h and Supplementary Fig. 1c). It is not clear whether this observation reflects insertion preferences away from exons or stronger and more dominant deleterious effects of type B insertions within exons, which would thus be rapidly purged and therefore rarely detected even at low frequency.

The deficit of HF TE insertions within exons (type A) or introns (types A&B) is not compensated uniformly across other categories. Rather, the increased proportion of HF TE insertions was most pronounced in the 5′ or 3′ UTRs of genes for both types of TE superfamilies (+4.5% and +2.6% respectively, Fig. 1f and Supplementary Fig. 1c). Although we cannot rule out alternative hypotheses, this observation suggests that TE insertions located within these two particular genic compartments tend to be under positive rather than purifying selection.

### Polysomic masking causes TE over-accumulation in tetraploids

**Fig. 2:**
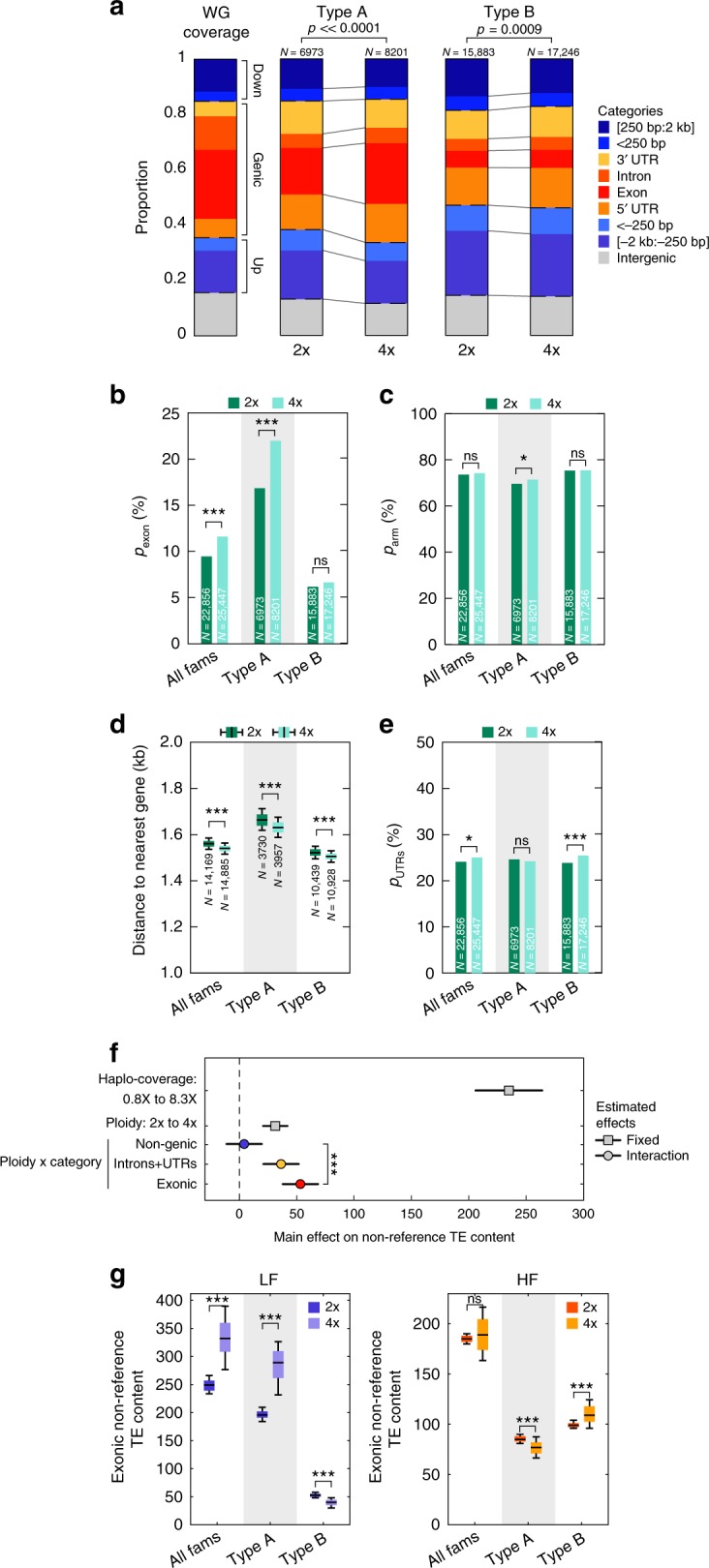
Increased exonic TE load from relaxed purifying selection in autotetraploids. **a** Distribution of TE insertions across categories of insertions for type A and type B superfamilies in diploids and tetraploids compared to reference genome annotations with *p*-values of *χ*^2^-test. **b** Fraction, *p*_exon_, of TE insertions overlapping exons for all, type A, and type B superfamilies in diploids and tetraploids with *p*-values of *χ*^2^-test. **c** Fraction, *p*_arm_, within chromosome arms (>5 Mb away from centromeres) of TE insertions for all, type A, and type B superfamilies in diploids and tetraploids with *p*-values of *χ*^*2*^-test. **d** Boxplots of distance to nearest gene of non-genic TE insertions for all, type A, and type B superfamilies in diploids and tetraploids across 1000 bootstraps and *p*-value of *t*-test between ploidies. Boxplot center lines, median; box limits, upper and lower quartiles; whiskers, 9th and 91st quantiles. **e** Fraction, *p*_UTRs_, of TE insertions overlapping UTRs (5′ and 3′) for all superfamilies, type A, and type B TE superfamilies in diploids and tetraploids with *p*-values of *χ*^2^-test. **f** Estimated MLM effects and interaction terms of haplo-coverage, ploidy, and category of insertions (non-genic, introns and UTRs, or exonic) on non-reference TE content. Horizontal lines indicate 95% confidence intervals for each effect value. *p*-values for each coefficient corresponds to the *t*-statistic of the hypothesis test that the corresponding coefficient is equal to zero or not. **g** Number of TE insertions within exons carried by 100 individuals for all, type A, and type B superfamilies in diploids and tetraploids at low-frequency (LF, left panel) and high-frequency (HF, right panel) with standard deviations across 100 random samples and *p*-value of *t*-test between ploidies. Boxplot center lines, median; box limits, upper and lower quartiles; whiskers, 9th and 91st quantiles. *p* < 0.001: ***; *p* < 0.01: **; *p* < 0.05: *; *p* ≥ 0.05: ns. The source data underlying Fig. 2a–e are provided as a Source Data file.

In contrast to diploids, autotetraploids (i.e. 4x) show a marked increase in the proportion (*p*_exon_) of type A insertions within exons (*χ*^2^-test *p* < 0.001, Fig. 2a, b and Supplementary Fig. 2a). In addition, type A insertions are slightly more frequent within chromosome arms (*χ*^2^-test *p* < 0.05, Fig. 2c and Supplementary Fig. 2b) and closer to genes (0.10 kb, *t*-test *p* < 0.001, Fig. 2d and Supplementary Fig. 2c) in the autotetraploids relative to diploids. The proportion (*p*_UTRs_) of type A insertions within UTRs are mostly unaffected by ploidy (Fig. 2e). In contrast, type B insertions showed few differences between ploidies, except for a small increase in their proportion within UTRs in the tetraploids (Fig. 2e), the significance of which is unclear. Thus, outside of type A exonic insertions, differences between ploidies remain limited. Moreover, the overall carrier-frequency spectra did not significantly differ (Supplementary Fig. 3a) despite potential biases affecting differently the detection of TE insertions in autotetraploids (see Methods). Combined, these observations confirm that the TE landscapes captured in diploids and tetraploids are globally similar, in line with the relatively recent origin of the *A. arenosa* autotetraploids^16^, and are consistent with purifying selection being significantly relaxed on genic TE insertions in the autotetraploids.

In keeping with this scenario, when corrected for coverage per haploid genome (haplo-coverage) tetraploids tend to harbor a higher number of TE insertions than their diploid counterparts (see Methods, Fig. 2f, Supplementary Table 2) and this increase is predominantly contributed by exonic TE insertions. Furthermore, tetraploids on average carry ~30% more low-frequency TE insertions (mostly type A) within exons than diploids (see Methods), but the number of high-frequency TE insertions is similar between diploids and autotetraploids (Fig. 2g and Supplementary Fig. 2d). Given that autotetraploids arose relatively recently^16^ and are therefore unlikely to have reached their mutational balance equilibrium^22^, we can indeed expect the relaxation of purifying selection to be mostly visible over low-frequency TE insertions, as these tend to be young and not shared with diploids.

**Fig. 3:**
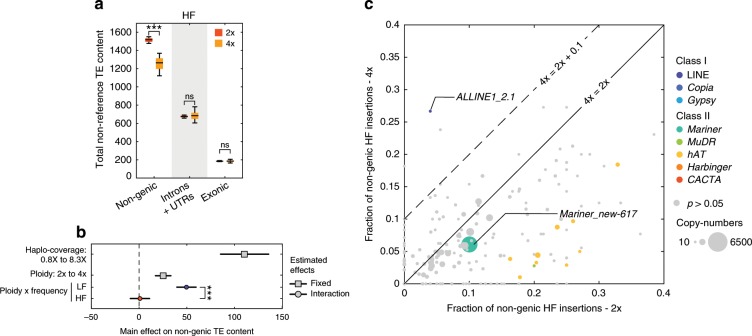
Absence of genome-wide or family specific transposition burst hallmarks in autotetraploids. **a** Number of TE insertions carried by 100 individuals by category (non-genic, introns and UTRs, exonic) in diploids and tetraploids at high-frequency (HF) with standard deviations across 100 random samples and *p*-value of *t*-test between ploidies. Boxplot center lines, median; box limits, upper and lower quartiles; whiskers, 9th and 91st quantiles. **b** Estimated MLM effects and interaction effects of haplo-coverage, ploidy, and insertion frequency on non-genic TE content. Horizontal lines indicate 95% confidence intervals for each effect value. *p*-values for each coefficient corresponds to the *t*-statistic of the hypothesis test that the corresponding coefficient is equal to zero or not. **c** Fraction of non-genic insertions at high-frequency (HF) in tetraploids versus diploids by TE family. TE families with *χ*^2^ *p*-values < 0.05 are colored. *p* < 0.001: ***; *p* < 0.01: **; *p* < 0.05: *; *p* ≥ 0.05: ns. The source data underlying Figs. 3a and 3c are provided as a Source Data file.

According to the genome-shock hypothesis^9^, the global increase in TE content observed in the autotetraploids could also be the footprint of a general transposition burst at the time of the WGD event. Under this scenario, tetraploids compared to diploids should exhibit an excess of high-frequency TE insertions, especially when non-genic, as these are the least exposed to purifying selection (Fig. 1h). This is clearly not the case, as tetraploids carried less non-genic high-frequency TE insertions than diploids, but similar amounts in genes (Fig. 3a, see Methods). After correction for haplo-coverage, we observed an excess of non-genic TE insertions in the tetraploids at low-frequency only (Fig. 3b, Supplementary Table 3), all but ruling out an ancestral genome-wide transposition burst. Nonetheless, 35% of high-frequency non-genic TE insertions in the autotetraploids are not present in the diploids (Supplementary Fig. 3b), suggesting that they could have originated from TE-family-specific transposition bursts. To explore this possibility, we compared the proportion of high-frequency non-genic TE insertions between tetraploids and diploids for the 201 TE families with enough non-reference copies (>10) in each of the two ploidy groups and found that no TE family shows a significant excess (*χ*^2^, *p* < 0.05) of high-frequency non-genic TE insertions in the autotetraploids, with one possible exception (*ALLINE1_2.1*; *p* = 0.036, Fig. 3c).

Collectively, our data indicate that the increased TE content in autotetraploids results mainly if not exclusively from the progressive accumulation of TE insertions within genes, especially exons, thanks to relaxed purifying selection, rather than from any appreciable transposition burst.

### *Copia* over-accumulation offers variants for local adaptation

**Fig. 4:**
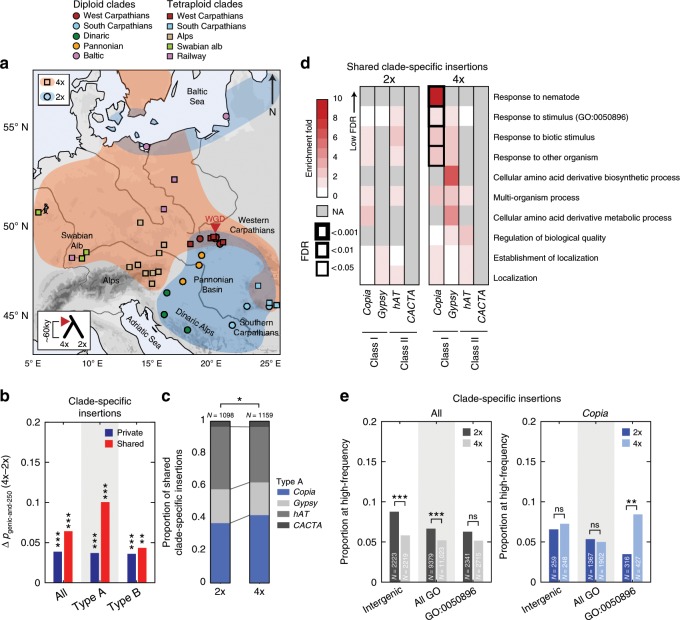
Local positive selection on *Copia* genic insertions in tetraploids. **a** Map of the tetraploid and diploid populations colored by clade. **b** Difference between tetraploids and diploids of the proportion of genic and near-genic (<250 bp) insertions present in only 1 clade (clade-specific) and private or shared within the clade for all, type A, and type B superfamilies with *p*-values of *χ*^2^ test between ploidies (type A: 2x *n* = 4597 private and 1098 shared, 4x *n* = 5524 private and 1159 shared; type B: 2x *n* = 8921 private and 2563 shared, 4x *n* = 9646 private and 2335 shared). **c** Proportion among shared clade-specific type A insertions from each type A superfamily in diploids and tetraploids with *p*-value of *χ*^2^ test between ploidies. **d** GO enrichments in diploids and tetraploids among genes carrying or nearby shared clade-specific insertions for type A TE superfamilies. **e** Proportion in diploids and tetraploids of clade-specific insertions at locally high frequencies (shared by three or more individuals) within intergenic regions, within or near genes, or within or near stimulus response genes (GO:0050896) for all superfamilies or only *Copia* insertions with *p*-values of *χ*^2^ test between ploidies*. p* < 0.001: ***; *p* < 0.01: **; *p* < 0.05: *; *p* ≥ 0.05: ns. The source data underlying Fig. 4b–e are provided as a Source Data file.

Given the association of polyploidy with colonization potential^27–29^, we asked whether the increased TE content within or near genes could contribute to local adaptation of autotetraploids. To this end, we first identified clade-specific TE insertions for the two ploidy groups (See Methods, Fig. 4a) and found a significant enrichment in tetraploids compared to diploids within or near (<250 bp) genes, which is most pronounced for local high-frequency type A insertions (Fig. 4b and Supplementary Fig. 4a). This finding suggests therefore that type A insertions within or near genes are commonly under local positive selection in tetraploids, unlike those of type B (Fig. 4b). Further analysis of these local high-frequency type A insertions indicated that they are specifically enriched for *Copia* LTR-retrotransposons in tetraploids (Fig. 4c). Consistent with observations in *A. thaliana* and other plant species^30^, this TE superfamily shows in both ploidies a marked insertion preference, visible at low-frequency, towards genes responsive to biotic stimuli (Supplementary Fig. 4b), a class of genes that are typically under diversifying selection^31,32^. Moreover, *Copia* insertions within or near such genes are significantly enriched at local high-frequency only in tetraploids (Fig. 4d) and are more likely to reach high frequencies (three or more individuals within a clade, HF3) than all other TE insertions (Fig. 4e), independently of the longer size of this category of genes (Supplementary Fig. 4c). Collectively these findings indicate that *Copia* insertions within environmentally responsive genes are more frequently under local positive selection specifically in tetraploids.

**Fig. 5:**
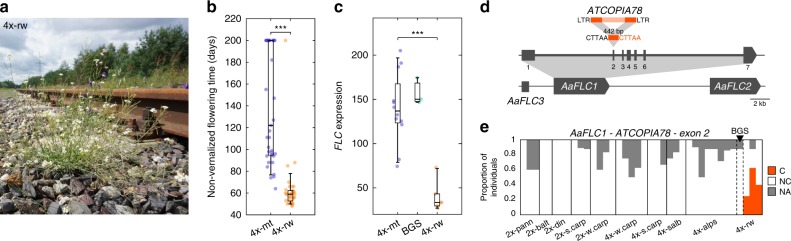
Railway-specific *ATCOPIA78* solo-LTR exonic insertion within *FLC*. **a** Picture of a tetraploid *A. arenosa* individual growing on railway ballasts near Świnoujście, Poland, in July 2017. **b** Non-vernalized flowering time, measured in days to the first open flower, of mountain (4x-mt) and railway (4x-rw) tetraploid populations. Data was redrawn from Baduel et al.^20^. Boxplot center lines, median; box limits, upper and lower quartiles; whiskers, 9th and 91st quantiles. **c** *FLC* expression tetraploid populations from mountain (4x-mt) and railway (4x-rw) and the hybrid mountain-railway population BGS. Data were redrawn from Baduel et al.^35^. Boxplot center lines, median; box limits, upper and lower quartiles; whiskers, 9th and 91st quantiles. **d** Map of *FLC* region with 442bp-long *ATCOPIA78* solo-LTR insertion in 2nd exon of *AaFLC1* identified by fosmid sequencing. **e** Proportion of individuals across populations by clade (pann pannonian, balt baltic, din = dinaric, s.carp = south-carpathians, w.carp = west carpathians, salb = swabian alb, rw = railway) carrying (C) or not-carrying (NC) the *ATCOPIA78* solo-LTR insertion or not-assigned (NA). Hybrid mountain-railway population BGS is indicated with black triangle. The source data underlying Fig. 5e are provided as a Source Data file.

One clear example of local adaptation of *A. arenosa* tetraploids to a novel habitat is provided by the successful colonization by one lineage of railway ballasts (Fig. 5a) across central Europe. In previous studies, we have shown that adaptation to the harsher railway environment is associated with a switch to early flowering and a loss of expression of the floral repressor gene *FLC*^20,21^(Fig. 5b, c). *FLC* is a known hotspot for *Copia* insertions in *A. thaliana*^25^, so we investigated whether railway tetraploids may be carrying such an insertion. Due to local syntenic divergences between *A. arenosa* and *A. lyrata* in the *FLC* region^33^, we could not use our *A. lyrata-*based pipeline to search for the presence of non-reference TE insertions within the two full length *A. arenosa FLC* paralogues (*AaFLC1* & *AaFLC2*). Instead, we sequenced three fosmid clones of the *FLC* region that were obtained from an early flowering railway individual. Comparison to the BAC sequence of the *FLC* region that was obtained previously from a late flowering individual^33,34^, revealed a number of intergenic or intronic structural variants. Notably, two clones contained the same *ATCOPIA78* solo-LTR insertion in the 2nd exon of *AaFLC1* (Fig. 5d), which is the main contributor (>80%) to total *FLC* expression in *A. arenosa*^20^. Examination of whole-genome sequencing data of the 286 *A. arenosa* accessions for the presence of this solo-LTR insertion (see Methods) indicated that it is present in the three railway tetraploid populations, but not in any of the 15 diploid populations nor in any of the other 19 tetraploid populations analyzed (Fig. 5e). It was also absent from a hybrid mountain-railway population, which, unlike the other railway tetraploids, is characterized by high *FLC* expression and was shown previously to be early flowering because of a specific allele of *CONSTANS*, another major flowering-time regulator^21^. Although further work is required to prove the functional impact of the *Copia* insertion, the complete association with the loss of *FLC* expression supports the notion that *Copia* retrotransposons generate alleles that can enable rapid adaptation to novel habitats.

The ability of tetraploids to absorb locally adaptive variants through introgression with local diploids has been often argued to favor their potential for rapid adaptation^22,35^. At the TE level, we could confirm the strong admixture between southern Carpathians diploids and tetraploids (Supplementary Fig. 5a). Yet, even in this case, diploid TE insertions represented only a minor fraction of TE insertions at local high-frequency (Supplementary Fig. 5b) which are, in the case of *Copia* in particular, almost exclusively locally sourced within the south Carpathian tetraploids.

## Discussion

Here, we performed a comprehensive analysis of TE dynamics in a natural diploid-autopolyploid species, which enabled us to assess the consequences of WGD without the confounding effect of hybridization. Our results indicate a major over-accumulation of TEs specifically within or near genes in the autotetraploids compared to their diploids counterparts, which supports the hypothesis that polysomy shelters TE insertions from selection. Conversely, we found no evidence of transposition bursts, neither genome-wide nor family-specific that could have been associated with the ancestral WGD event, which suggests that genome duplication alone, when not coupled with hybridization as in allopolyploids (e.g. tobacco^36^, rapeseed^37^), is insufficient to cause a severe “genomic stress”^38^. Finally, we present data supporting a role in local adaptation of the genic TE variants accumulating in tetraploids, including within a major adaptive locus.

Our conclusion that purifying selection in autotetraploids is relaxed confirms previous work based on single nucleotide polymorphisms (SNPs)^22^. However, given the young age of *A. arenosa* tetraploids^16^, the increase in mutational load generated by SNPs remains subtle^22^ whereas the impact of relaxing purifying selection is magnified for TE insertions, which often produce major-effect alleles contrary to SNPs. Over the long term, we expect this fundamental difference to be further exacerbated in a chain reaction-like manner as has been observed in *A. thaliana* TE-mutation-accumulation lines^30^. In fact, transposition rates should rapidly increase in tetraploid genomes as more active copies are tolerated and intergenic space gradually widens, increasing further the genome tolerance for more transposition events.

In turn, the progressive over-accumulation of genic TE variants caused by relaxed purifying selection could provide raw material for local adaptation of some tetraploid populations. Indeed, *Copia* insertions within or near stimulus response genes appear to be under local positive selection specifically in tetraploids. In contrast, interploidy admixture with local diploid populations contributes very few potentially adaptive *Copia* insertions.

The successful invasion of railway ballasts by *A. arenosa* tetraploids has been particularly intriguing as it represents a textbook example of the increased invasion potential often associated with polyploidy^27–29,39^, yet appears  to rely, at least in part, on diploid alleles imported through admixture^21^. Here, however, we found that a tetraploid-specific *Copia* exonic insertion within *AaFLC1* was exclusive to and shared by all of the rapid-cycling railway populations, with the exception of one hybrid population where early flowering was not associated with a loss of *FLC* expression^21^. Further work is needed to characterize case by case the phenotypic impact of local TE insertions in particular at the *FLC* locus.

While TEs may provide adaptive opportunities, as examples  in  *A. thaliana*^40^ as well as in *Drosophila*^41,42^ suggest, their mobilization is likely to generate predominantly either neutral or deleterious alleles. In addition, TEs are major contributors of spurious recombination events^9^, a key step in the “polyploid drop”^43^ that has historically almost inevitably followed WGD events^3,44^. Thus TE accumulation in autopolyploids may provide transient windows of adaptive opportunity but could eventually also lead to their evolutionary demise or at least their return to a diploid state.

In summary, our study sheds new light on the dynamic interactions between ploidy and the TE landscape with major implications for the adaptive potential and evolution of polyploids.

## Methods

### Data sources

Whole-genome sequencing data of *A. arenosa* individuals and paired-end alignments on the *A. lyrata* reference genome^22,23^ were obtained from Monnahan et al.^22^ where the phylogenetic relationships between the different clades of diploids and tetraploids (Fig. 4a) are described and analyzed.

### Identification of non-reference TE insertions

Split and discordant reads were extracted from individual alignments (2.8–18.1X, average 8.4X) and mapped on a joint TE library assembled from the annotation of all TEs in the *A. lyrata* reference genome (87,477 TEs^26^). Mapped reads were then soft-clipped and re-mapped to the reference genome to define putative non-reference TE insertion sites. These sites were intersected across individuals to identify those shared and supported in at least one individual by a minimum of three reads, including at least one upstream and one downstream. Negative coverage, as defined by the minimum read depth over the upstream and downstream boundaries of a putative TE insertion site, was then calculated for each individual across all putative TE insertion sites. To limit false-negatives, non-carrier individuals with less than five reads negative coverage or more than 100 (>10 times the average read depth) were considered as missing information or NA. Sites with more than 10 NA diploids and 10 NA tetraploids were considered non-informative for further analyses, resulting into 43,176 informative TE insertion sites (Supplementary Data 1). Due to the high heterozygosity of *A. arenosa*, in particular in tetraploids, we were not able to distinguish between homozygous and heterozygous TE insertions based on positive and negative coverage. Common TE names were obtained by intersection with TE annotation available in Pietzenuk et al.^45^ (MOESM3), or indicated as *superfamily_new-* followed by a unique identifier when previously unannotated (e.g. *Mariner_new-617*). Multiple TEs with the same common name were distinguished by a numbered suffix (e.g. *ALLINE1_2.1)*.

### Analyses of TE landscapes

Statistical analyses were performed using MATLAB (MathWorks, Inc., Natick, Massachusetts, United States). Carrier frequency was calculated at each TE insertion site over all non-NA individuals for the site. In diploids, 95% of TE insertions were at carrier-frequencies (*f*) under 12.2%. Under Hardy–Weinberg equilibrium, if *f* is below 12.2%, it should reflect allelic frequencies (*p*) below 6.3% (from the formula (1−*p*)^2^ = 1−*f*). Thus, >97.8% of diploid carriers are expected to be heterozygous, i.e. *p*~*f*/2. Similarly, in tetraploids at similar carrier-frequencies we should expect >96.5% of carriers to be simplex heterozygotes, i.e. *p*~*f*/4. This would suggest that at a given carrier frequency, corresponding allelic frequencies are lower in tetraploids than in diploids (by half). However, the coverage per haploid genome is also divided by 2 in tetraploids (thus ranging from 0.8X to 4.5X), which reduces the sensitivity of detection of simplex heterozygotes especially at low frequencies. This bias would lead, on the opposite, to an underestimation of allelic frequencies from carrier-frequencies in tetraploids.

As we were not able to distinguish the zygosity of the carriers of TE insertions, and thus to establish unbiased estimates of allelic frequencies for both ploidies, we performed cross-ploidy comparisons based on carrier-frequencies instead. Indeed, the carrier-frequency spectrum (calculated over random samples of 100 individuals per ploidy corresponding to 1% frequency resolution) did not significantly differ between the two ploidies (Supplementary Fig. 3a). Carrier-frequency thresholds for LF and HF TE insertions were calculated as the 10% and 90% percentile of the diploid frequency spectrum (≤1.2% vs ≥8.3%, respectively), and the same thresholds were used in tetraploids.

Densities of TE insertions across chromosomes were calculated by 100 kb windows and smoothed using a LOWESS (Locally Weighted Scatterplot Smoothing) regression for plotting (Fig. 1c, d). Categories of TE insertions were based on the RNAseq-improved annotation of the *A. lyrata* reference genome^23,46^. In case of ambiguity the following priority order was given: 3′UTR-5′UTR-exon-intron- < 250 bp upstream- < 250 bp downstream. For TE insertions both <2 kb upstream of one gene and <2 kb downstream of another gene the attribution to one of the two categories was defined by the closest gene. The representations of these categories in the reference genome (WG coverage in Fig. 1h) were obtained following the same rules and were then normalized by their average coverage calculated in 10 kb windows using a high-coverage diploid alignment (47X). Pairwise differences in proportions by category (either LF vs HF or 2x vs 4x) were estimated using a 2 × 2 *χ*^2^-test. Sequencing coverage is higher in tetraploids compared to diploids: 3.3–18.1X (average 8.7X) versus 2.8-16.5X (average 7.9X), but lower when expressed per haploid genome (haplo-coverage). As most TE insertions are likely heterozygotes (see calculations above) the sensitivity of the SPLITREADER is expected to be lower in tetraploids compared to diploids. Therefore, multiple linear models (MLMs) of individual non-reference TE content (Figs. 2f–3b) were obtained by stepwise multiple linear regressions using as parameters haplo-coverage (average coverage by haploid genome), ploidy, and category of TE insertion (non-genic, exonic, and other genic) for Fig. 2f, or haplo-coverage, ploidy, and carrier frequency (LF and HF) for Fig. 3b (Supplementary Tables 1, 2). Fixed effects or interaction terms were added or removed based on the *p*-value for an *F*-test of the change in the sum of squared error with or without the term. Average TE content by ploidy was calculated based on 100 subsamples of 100 individuals by ploidy. Error-bars indicate standard deviation observed across 100 samples and statistical differences were obtained by 2-way *t*-tests (Figs. 2g–3a). Gene Ontology (GO) enrichments were calculated based on genes <250 bp away from TE insertions of interest using agriGO in comparison with *A. thaliana* reference annotation (http://bioinfo.cau.edu.cn/agriGO/).

### RNAseq analysis

RNAseq datasets were obtained from previously published datasets^21,22^ for a subset of six tetraploid and three diploid populations with three individuals each. For each gene, we compared the average expression levels in populations where the nearest TE insertion was detected (carrier, C populations) to populations where the nearest TE insertion was not detected (non-carrier, NC). For genes with TE insertions >2 kb away (intergenic) C/NC ratio distribution was compared to randomly picked carriers and non-carriers populations (Supplementary Fig. 1a, Kolmogorov–Smirnov test). For genes with TE insertions <250 bp away (near-genic TE insertions), the distribution of C/NC ratios was compared to both random (KS; *p* < 5e−10) and intergenic (Supplementary Fig. 1b, KS *p* < 5e−3).

### Fosmid libraries

Because the *FLC* region in *A. arenosa* contains 3 *FLC* paralogues (two entire copies *AaFLC1* & *AaFLC2* and one truncated *AaFLC3*), that arose independently from the duplicated *A. lyrata FLC* paralogues^33^, Illumina short-reads align poorly on the FLC locus of *A. lyrata* reference genome impairing the detection of non-reference TE insertions. Instead, we extracted DNA from three-week-old plants from a mainland railway population (TBG) using a large-scale CTAB protocol including treatment with pectinase. We constructed a fosmid library using the Copy Control Fosmid Library Production Kit (Epicentre) and screened it using DIG-labeled (Roche) PCR probes to the center of the *FLC* locus (primers 5′ AGTGTAACTTCAATGGCAGAAAACCCT 3′ and 5′ ATGTGGCGGTAAGCAGAGATGACC 3′). We bar-coded positive clones and sequenced 100 bp paired-end reads on an Illumina HiSeq 2000. We aligned *FLC* reads (which performed poorly in de novo assembly) to an *A. arenosa* BAC (GenBank accession no. FJ461780) using BWA. We identified insertions and deletions by targeted de novo alignment using Velvet^47^. The 442 bp-long insertion present in the second exon of *AaFLC1* in two out of three fosmids bore a 94.5% similarity with the LTR sequence of *ATCOPIA78* (RepeatMasker blast) in addition to a 5 bp tandem site duplication (TSD), as expected from *Copia* insertions^48^.

We detected carrier individuals for this *Copia* insertion from whole-genome resequencing data by re-mapping paired-end reads to two versions of an updated BAC sequence^20^ including or not the TE insertion. We considered as carrier any individual with at least 1 read bridging the insertion extremities by at least 20 bp, and as non-carrier when no read bridged insertion extremities and at least 4 reads bridged the TSD.

### Reporting summary

Further information on research design is available in the Nature Research Reporting Summary linked to this article.

## Supplementary information

Supplementary Information

Peer Review File

Reporting Summary

Description of Additional Supplementary Files

Supplementary Data 1

## Data Availability

Whole-genome resequencing data of Monnahan et al.^22^ are deposited in the Sequence Read Archive with the primary accession code PRJNA484107 and PRJNA472485 for RNAseq data. Data supporting the findings of this work are available within the paper and its Supplementary Information files. A reporting summary for this Article is available as a Supplementary Information file. The datasets generated and analyzed during the current study are available from the corresponding authors upon request. The source data underlying Figs. 1b-h, 2a-e, 2g, 3a, 3c, 4b-e, and 5e, and Supplementary Figs. 1–5 are provided as a Source Data file.

## Source data

Source Data

## Code availability

The SPLITREADER^25^ code is freely available at https://github.com/LeanQ/SPLITREADER, Bowtie2 v2.3.2 at https://sourceforge.net/projects/bowtie-bio/files/bowtie2/2.3.5.1/, Picard Tools at https://broadinstitute.github.io/picard/, SAMtools V1.2.1 at http://www.htslib.org/download/, and MATLAB version 9.3.0 (R2017b) from the MathWorks Inc. https://www.mathworks.com/.
