# Supplementary material for: Relaxed purifying selection in autopolyploids drives transposable element over-accumulation which provides variants for local adaptation
Source: Nat Commun. 2019 Dec 20;10:5818. doi: 10.1038/s41467-019-13730-0 (PMC6925279; doi:10.1038/s41467-019-13730-0)
Supplement: Supplementary file 1 — Supplementary Information [file 41467_2019_13730_MOESM1_ESM.pdf]

Relaxed purifying selection in autopolyploids drives transposable element over-accumulation which provides variants for local adaptation

Baduel *et al.*

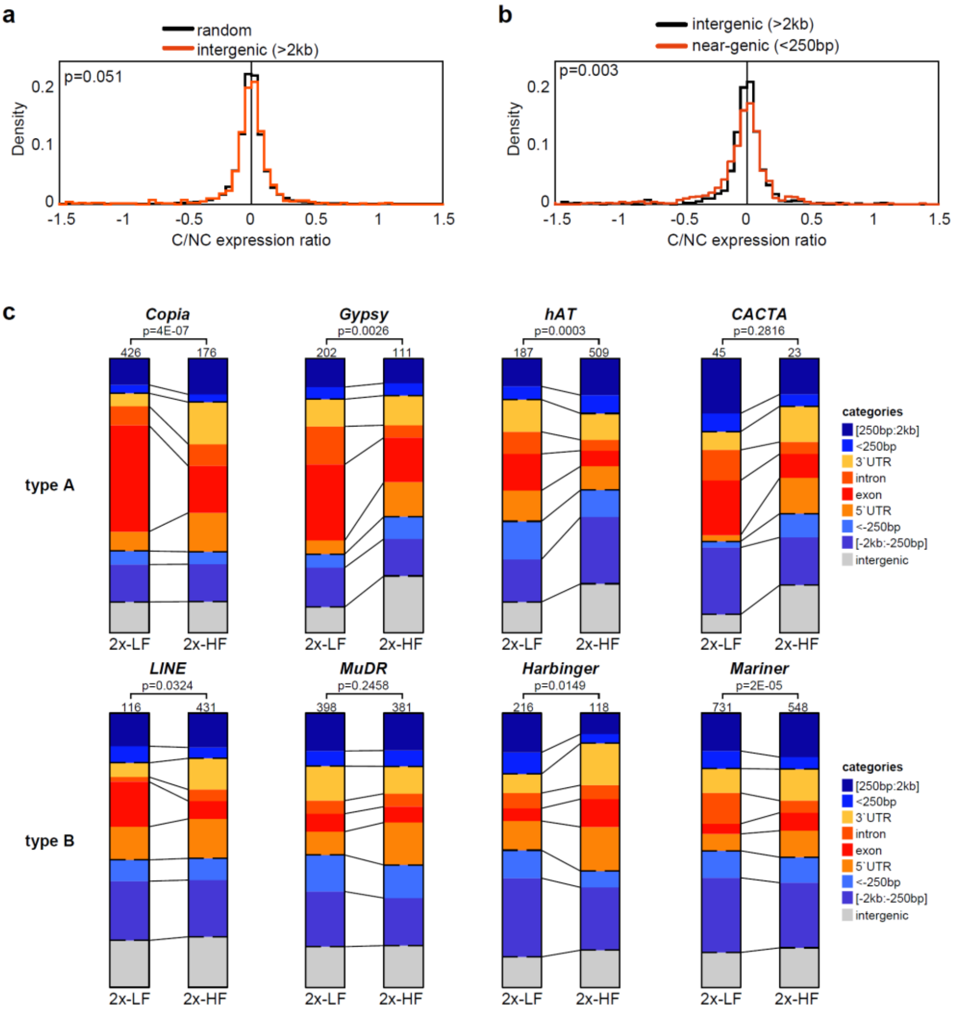

Supplementary Figure 1. Purging of genic and near-genic TE insertions in diploids. (a) Distribution of expression log-ratios of genes without any non-reference TE insertion detected within 2kb between carriers (C) and non-carriers (NC) of the nearest intergenic TE insertion (>2kb away) compared to random assignment of carriers and non-carriers acrosss all genes. p-value of Kolmogorov-Smirnov test. (b) Distribution of C/NC log-ratios of genes with a non-reference TE insertion detected within 250bp between carriers (C) and non-carriers (NC) of the nearest TE insertion (<250bp away) compared to genes without any non-reference TE insertion detected within 2kb (intergenic). p-value of Kolmogorov-Smirnov test. (c) Distribution of low- and high-frequency non-reference TE insertions in diploids across categories of insertions for all TE-superfamilies with p-values of χ^2 test. Source data are provided as a Source Data file.

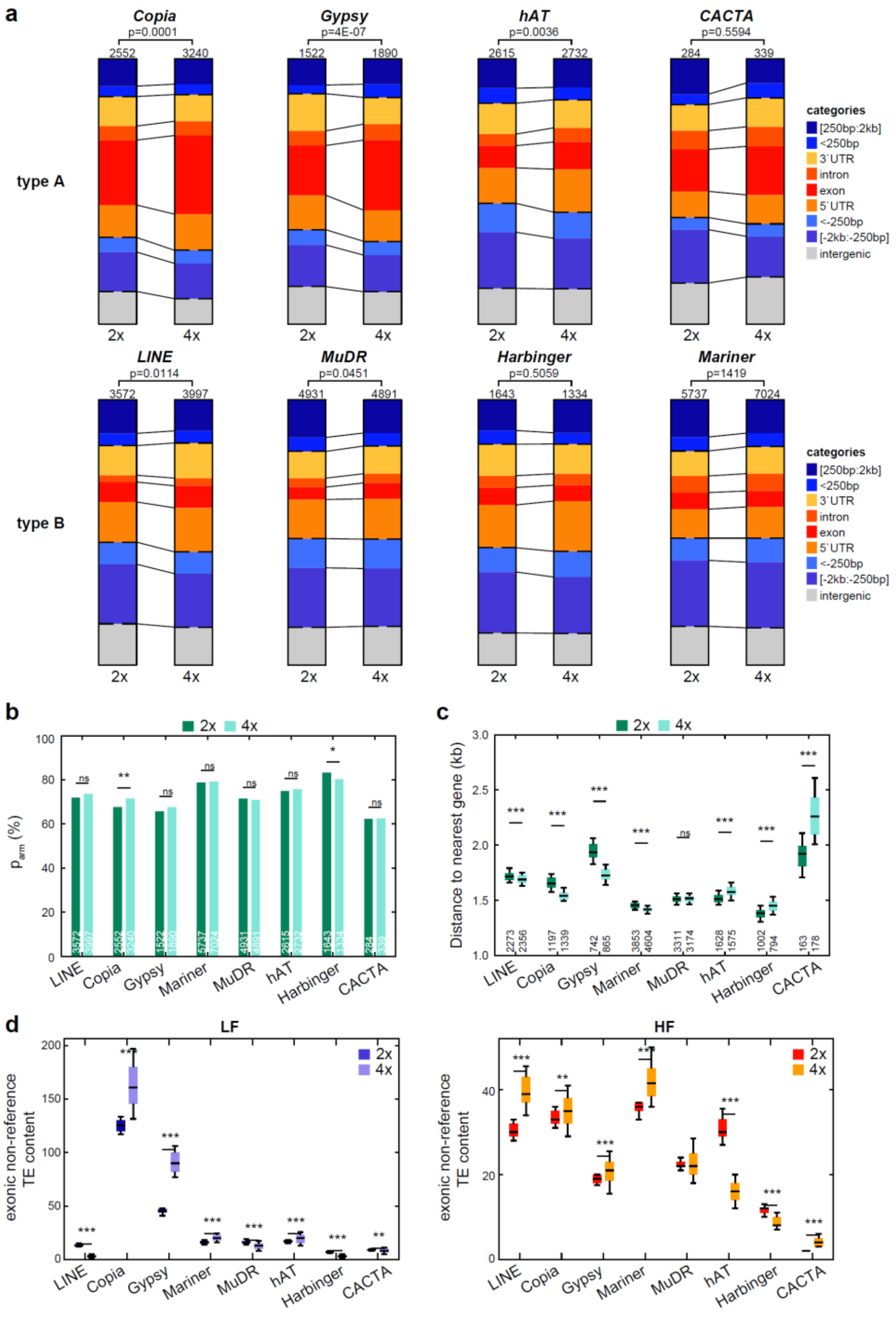

Supplementary Figure 2. Increased genic and near-genic TE load in tetraploids. (a) Distribution of non-reference TE insertions in diploids and tetraploids across categories of insertions for all superfamilies individually with p-values of χ² test. (b) Fraction, parm, within chromosome arms (>5Mb away from centromeres) of non-reference TE insertions for all superfamilies individually in diploids and tetraploids with p-values of χ² test. (c) Distance to nearest gene of non-reference TE insertions for all superfamilies individually in diploids and tetraploids with p-value of t-test between 1000 bootstraps. Boxplot center lines, median; box limits, upper and lower quartiles; whiskers, 9th and 91st quantiles. (d) Number of non-reference TE insertions within exons carried by 100 individuals for all superfamilies individually in diploids and tetraploids at low-frequency (LF, left panel) and high-frequency (HF, right panel) with p-value of t-test between both 100 random samples. ($p<0.001$: ***; $p<0.01$: **; $p<0.05$: *; $p\geq0.05$: ns). Source data are provided as a Source Data file.

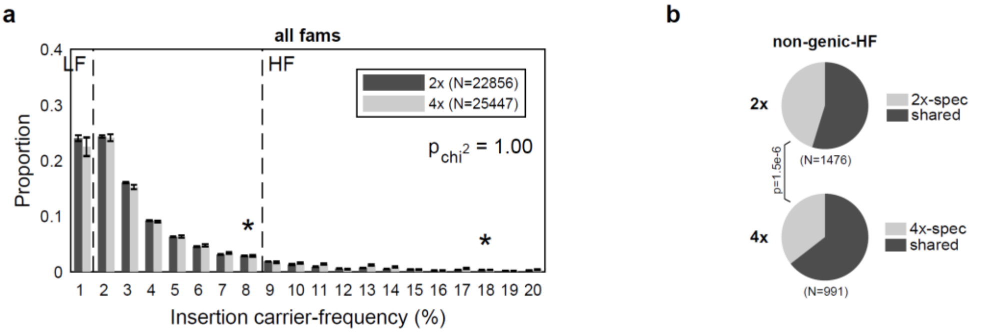

Supplementary Figure 3. Frequency spectrum comparisons between diploids and tetraploids. (a) Carrier-frequency spectrum averaged over 100 samples of 100 diploids and 100 tetraploids (1% resolution). Error bars represent the standard deviation between 100 samples, and stars indicate bins with p-value of t-test <0.05 between diploids and tetraploids. p-value of overall χ^2 test between the two distributions is also provided. (b) Proportion of non-genic HF TE insertions within diploids (2x) and tetraploids (4x) that are either ploidy-specific or shared with the other ploidy. p-value is calculated using a χ^2 test. Source data are provided as a Source Data file.

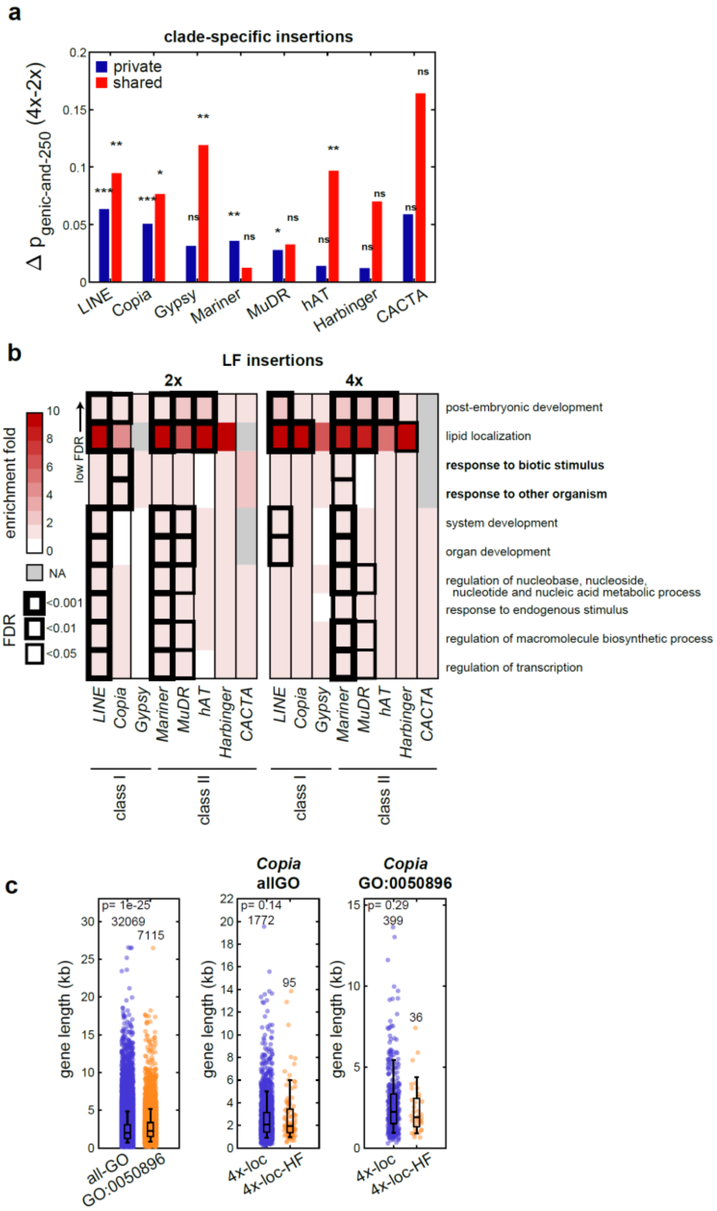

Supplementary Figure 4. Retention of genic and near-genic clade-specific TE insertions in tetraploids. (a) Difference between tetraploids and diploids of the proportion of genic and near-genic (<250bp) insertions present in only 1 clade (clade-specific) or shared within the clade for all superfamilies individually with p-values of $\chi 2$ test between ploidies. (b) GO enrichments in diploids and tetraploids among genes carrying or nearby (<250bp) LF insertions for all TE superfamilies (c) Distribution of gene length between all GO and stimulus response genes (GO:0050896) (left panel), between genes carrying or nearby (<250bp) Copia insertions in tetraploids that are clade-specific (4x-loc) or clade-specific and at high-frequency (4x-loc-HF) within all GO (middle panel) or within GO:0050896 only (right panel). p-values of pairwise t-test are indicated for each panel. p<0.001: ***; p<0.01: **; p<0.05: *; p≥0.05: ns. Source data are provided as a Source Data file.

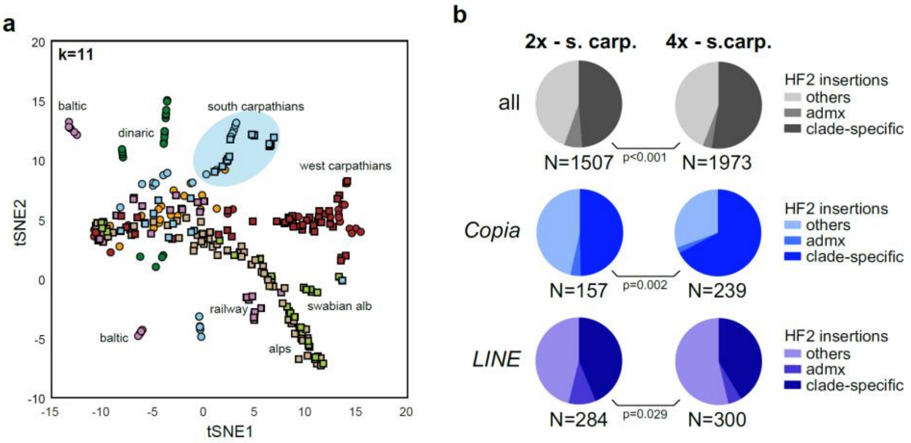

Supplementary Figure 5. Contribution of local admixture from diploids to tetraploids local high-frequency TE insertions in tetraploid clade. (a) tSNE clustering of A. arenosa individuals by shared TE variation after NNMF-reduction. (b) Proportions of high-frequency insertions (HF2, found in 2 or more individuals within a clade) in south Carpathian diploids or tetraploids that are clade-specific or admixed locally between ploidies (admx) for all TE superfamilies, Copia, or LINE insertions. Source data are provided as a Source Data file.

Supplementary Table 1. Non-reference TE content distribution within chromosome arms.

100kb region	2x-LF	2x-HF
gene-poor	70	109
gene-rich	204	110

Note: Counts of 2x-LF and 2x-HF non-reference TE insertions within gene-poor (lower decile of density of reference genes) vs gene-rich (upper decile) 100kb regions of chromosome arms.

Supplementary Table 2. Effect of TE insertion categories on increased tetraploid TE content.

	Estimate	SE	t-Stat	p-Value
(Intercept)	1.235	18.923	0.065265	0.94798
TEfeature_genic	-149.92	12.966	-11.563	8.0386E-29
TEfeature_exonic	-220.86	12.966	-17.033	3.3946E-56
Haplocoverage	63.868	4.1638	15.339	4.6159E-47
ploidy_4	114.46	23.352	4.9014	1.1398E-06
TEfeature_genic:ploidy_4	50.04	16.299	3.0701	0.0022078
TEfeature_exonic:ploidy_4	74.249	16.299	4.5555	5.9917E-06
Haplocoverage:ploidy_4	-36.86	6.8372	-5.3912	9.0744E-08
Number of observations	858			
Error degrees of freedom	850			
Root Mean Squared Error	94			
R-squared	0.493			
Adjusted R-squared	0.489			
F-statistic vs constant model	118			
p-value	6.14E-121			

Note: Estimated effects and interaction terms in stepwise multiple linear regression of haplo-coverage, ploidy, and insertion feature (exonic, intronic + UTRs, non-genic) on TE content. Linear regression model: insertion_Nb ~ 1 + TEfeature + Haplocoverage + ploidy + TEfeature*ploidy + Haplocoverage*ploidy

Supplementary Table 3. **Effect of TE insertion frequency on increased tetraploid TE content.**

	Estimate	SE	t-Stat	p-Value
(Intercept)	-71.701	8.3318	-8.6058	7.494E-17
Tefreq_HF	67.4	4.8554	13.882	6.43E-38
Haplocoverage	21.418	1.9096	11.216	1.673E-26
ploidy_4	78.849	10.266	7.6809	6.994E-14
TEfreq_HF:ploidy_4	-48.731	6.1033	-7.9845	7.912E-15
Haplocoverage:ploidy_4	-10.345	3.1356	-3.2992	0.0010305
Number of observations	572			
Error degrees of freedom	566			
Root Mean Squared Error	35.2			
R-squared	0.394			
Adjusted R-squared	0.89			
F-statistic vs constant model	73.7			
p-value	2.1E-59			

Note: Estimated effects and interaction terms in stepwise multiple linear regression of haplo-coverage, ploidy, and insertion frequency (LF, HF) on non-genic TE content. Linear regression model: exogenic_insertion_Nb ~ 1 + TEfreq + Haplocoverage + ploidy + TEfreq*ploidy + Haplocoverage*ploidy
