# Supplementary material for: Relaxed purifying selection in autopolyploids drives transposable element over-accumulation which provides variants for local adaptation
Source: Nat Commun. 2019 Dec 20;10:5818. doi: 10.1038/s41467-019-13730-0 (PMC6925279; doi:10.1038/s41467-019-13730-0)
Supplement: Supplementary file 4 — Description of Additional Supplementary Files [file 41467_2019_13730_MOESM4_ESM.docx]

**Description of Additional Supplementary Files**

File name: Supplementary Data 1
Description: the entire dataset of 43,176 TE insertions, their information (TE-name, TE-superfamily, common name), their position, and their presence / absence across the 286 individuals
