# Supplementary material for: Relaxed purifying selection in autopolyploids drives transposable element over-accumulation which provides variants for local adaptation
Source: Nat Commun. 2019 Dec 20;10:5818. doi: 10.1038/s41467-019-13730-0 (PMC6925279; doi:10.1038/s41467-019-13730-0)
Supplement: Supplementary file 2 — Peer Review File [file 41467_2019_13730_MOESM2_ESM.pdf]

Reviewers' comments:

Reviewer #1 (Remarks to the Author):

The manuscript submitted by Baduel et al. aims at deciphering the impact of autopolyploidy on TE-driven genome dynamics. The authors have exploited the genomic sequences of 300 Arabidopsis arenosa plants, among which 180 are polyploid.
As claimed by the authors, A. arenosa is a great system to study the short term genomme evolution of polyploidy because the origin of 4N A. arenosa is estimated to be ~ 60 ky. The comparative genomic approach between the diploid and tetraploid accessions is well done and its design is smart. The paper is well written, the science is sound and I only have minor critics to address to it :

- Throughout the manuscript, the authors are tempted to draw general conclusions from their results (a bad habit of arabidopsis genomicists indeed). I think they must at least temper their conclusions : a small genome is less prone to accumulate new insertions than a big one, simply because the probability that new insertions impede gene function is higher when the gene space is more prevalent....

- Even if the polyploidy is of recent origin, I think that it would be worthwhile to provide the reader with a phenetic tree of the the ~300 accessions used in this study. This can be very easily done using SNP data mined out from the raw seq.

- authors have ignored in the introduction all the references on tobacco and rapeseed.

- p4 l100-102 : "further analysis revealed that the deficit of HF insertions within or near genes is most pronounced for copia, gypsy CACTA and hAT TE superfamilies which show an apparent insertion preference for genes especially exons...." please rephrase. This sentence is an oxymoron.

- p7 l167 : in polyplids : "further analysis of these local HF type A insertions indicated that they are specifically enriched for Copia LTR." the authors bring forward the propensity of Copia elements to insert into genes responsive to biotic stimuli. This is not the case for the diploids (see above). please clarify this.

- Adaptation : the part on adaptation suffers over-interpretation. Association does not mean that there is a causal relationship between insertion and adaptation. The first step should be to conduct basic functional studies on this. Anyway, the link between early flowering and railway colonization is not obvious to a non specialist.

conclusion : this paper should be published in nature communications. I could share the enthousiasm of the authors for this study, but certainly could also encourage them to rephrase some of their conclusions.

Reviewer #2 (Remarks to the Author):

Review of “Transposable elements over-accumulation in autopolyploids results from relaxed purifying selection and provides variants for rapid local adaptation”

This work by Baduel et al uses a published dataset of short Illumina sequences among diploids and autotetraploids (thereafter polyploids) of Arabidopsis arenosa to characterize copies of transposable elements (TEs; most main types). As in prior work on this system, the authors here circumvent the necessity of comparing diploids to experimental and natural polyploids by relying on range-wide populations. Contrasting with prior work focused on SNP, emphasis on TEs here yields original insights

on possible causes and consequences of genome dynamics in polyploids. Given that limited work concluded on the extent at which whole-genome duplication (WGD) triggers or enables TE dynamics, such a study is of great interest and I read it with interest.
The manuscript is concise and well-written, although the presentation lacks of details to properly evaluate the significance of reported patterns. More comprehensive supporting information should generally be provided (see comments below). Further justification of patterns involving the polyploid dataset would enable proper interpretation in the light of comparative genomics as well as population genetics. With a slightly increased structure helping non-specialists to follow the line of thought, the study is likely to offer convincing insights on interactions between TEs and WGD in shaping genome evolution.

Main comments
1. L.49: A. arenosa should better introduced as to justify the assumption that the system enables to investigate "WGD independently of the confounding effects of hybridization". Given prior insights that the species hybridizes with Arabidopsis lyrata (Arnold et al. 2016 PNAS), I was here confused.
2. The characterization of "non-reference TE insertions" must be further described and validated. First, the use of the outgroup A. lyrata to identify polymorphic TE copies in A. arenosa should be further justified. It is here based on an unpublished TE annotation of the A. lyrata genome (a summary would be welcome) and, at least, how TE copies were grouped into families should be described. The here presented dataset otherwise remains loosely defined and possible biases difficult to assess.
3. Related to prior comment, provided specificities of TE calling, how individual sequencing coverage impacts on the accuracy of TE detection should be shown. How does it affect the call of presence vs absence in diploids as well as polyploids? How were heterozygotes treated in diploids vs polyploids (if not, this is to be stated transparently)? How was non-TE variation (e.g. indels) affecting the identification of homologous "TE insertions" taken into consideration in diploid vs polyploid individuals? Such clarification looks necessary to follow analyses and their interpretations.
4. The possible accumulation of non-reference TE copies along chromosome arms should be further justified in the light of possible biases against their detection across TE-rich chromosome regions. More specifically, how were pericentromeres here defined in A. arenosa should be briefly justified. Confirmatory comparisons of gene-rich vs gene-poor regions of chromosome arms may strengthen assessment of purifying selection on TE copies.
5. How were low-frequency (LF) were high-frequency (HF) TE copies defined should be better described. Note that Fig H-S2 and Fig F-H-S2 mentioned here were not accessible (not uploaded). Although the presented range-wide survey is reassuring, further justification of LF copies being recently inserted copies looks necessary (maybe on a subset of loci). In particular, L. l87 states that HF and LF are parameters of age when selection is not a factor, but L93 says that selection is a factor. Please, clarify.
6. Not only the use of an outgroup reference genome (comments 2 and 3), but also the focus on natural populations makes it inherently difficult to properly distinguish copies out of de novo insertion from segregating copies. Accordingly, I would recommend to avoid wording such as "insertion" (e.g. "insertion preference"; L. 102) as it creates confusion. For instance, "insertion sites" in A. arenosa (L. 256) could in fact be deletions in A. lyrata (used as reference). As a whole, loci under scrutiny should be carefully described to support clarified presentation also for non-specialists.
7. The distinction of 'type A' vs 'type B' superfamilies should be thoroughly presented. 'Type A' (Copia, Gypsy, hAT or CACTA) and 'type B' (LINE, Mariner, MuDR or Harbinger) include TEs using different transposition mechanisms, having different sizes, aso… Their possible unification must therefore be further justified. Why is hAT classified as 'type A'? Furthermore, as class I vs class II TEs may be expected to yield different patterns of "non-reference" copies (and transposons indeed show lots of non-reference copies here), such a clustering is surprising and should be further discussed; possibly regarding their timing of burst? Fig. 1h may suggest that type A proportions per category are similar to the whole genome proportions, contradicting the conclusion of "insertion preference for genes". Comparison of the observed number of non-reference TEs per category to their expectation based on the fraction of the genome occupied by each category could be provided for non-neutral insertion preference. Provided that the study relies heavily on the comparison of patterns between these

‘types’, such validation is crucial to follow corresponding results and offer a fully convincing paper.
8. Provided the use of an outgroup reference genome, the not-unexpected observation that TE copies “within or near (<250bp) genes but not further away” have an impact on their expressed should be further discussed. To what extent do issues related to micro-colinearity here affect such quantitative assessment? At least, this should be discussed in the light of prior survey in other Arabidopsis species that have reported longer-range effects (e.g. Holister et al. 2011. PNAS; Quadrana et al. 2016. Elife)
9. When it comes to the comparison of diploids and polyploids (L.120ff), the study would greatly benefit from a more detailed description of proxies used in polyploids as well as their subsequent comparison to diploids. How sequencing coverage affected the detection of copies and dosage, and how LF vs HF were assessed at the population level should be spelled out to demonstrate the absence of detection bias in polyploids.
10. In my understanding, non-reference TEs were independently called in diploids and polyploids, and thus include a large amount of shared copies among ploidy in both datasets. To what extent such data fit assumptions of subsequently used parametric tests should be further justified (e.g. L. 123, L. 139, L. 147). Statistics used to test patterns shown in different figures is not always crystal clear and, in particular, the legend of Fig.2 should be clear regarding chi-square vs t-tests. The correspondence between text and Fig. 2G and between Fig. 2B and Fig. 2G is obscure. Terms like parm, pexon, putrs should be properly defined in the text. More generally, as currently presented, effects seem rather low despite significant p-values. Provided that detection biases in polyploids can be ruled out, patterns tested (through e.g. resampling) among diploid-specific vs polyploid-specific copies may offer convincing insights.
11. Related to prior comment, Fig. 3c indicates that families (undefined; also see comment 2) show generally lower proportions of non-genic TE copies in polyploids as compared to diploids. In particular, it looks at odds with 35% of HF copies that are polyploid-specific (L. 147). This pattern remains confusing and possible underlying processes should be transparently discussed in the light of transposition vs frequency shifts (of LF vs HF) across ploidy levels.
12. Note that resampling of 100 accessions out of 105 diploid samples may be suboptimal to offer credible intervals. It should at least be justified.
13. Regarding the accumulation of Copia-related variants associated with local adaptation in polyploids, once issues of comment 9 have been clarified, population genetic structure should be further described. In particular, that enrichment of HF copies near genes of specific categories is not due to longer size of corresponding genes should be demonstrated to strengthen non-neutral claims. The mention of LF1, HF2 and HF3 across Fig 4 is confusing and should be further clarified.
14. From Fig. 4D, it may be thought that Gypsy not Copia are showing most significant patterns in polyploids or hAT in diploids, suggesting that the focus here on Copia must be further justified.
15. The detailed characterization of the FLC region is highly complementary, while interesting in itself. How conclusive insights were reached from short read data should be further justified in the light of (L. 182) “local syntenic divergence between A. arenoas and A. lyrata” at this locus. The legend of Fig 5B is missing several abbreviations

Reviewer #3 (Remarks to the Author):

This manuscript studied the TE dynamics following WGD based on ~300 individual Arabidopsis arenosa plants, including both diploids and autopolyploids. They identified 43,176 polymorphic TE insertions, and demonstrated that relaxed purifying selection rather than transposition bursts is the main driver of TE over-accumulation after WGD. In addition, TE insertion are enriched within or near abiotic and biotic stress response genes. Lastly, they showed TE insertion could affect a major flowering-time repressor gene and the mutation are correlated with the colonization of mainland railways. In general, this is a very interesting study about TE dynamics in diploid versus autopolyploid.

My comments:

1) With A. lyrata as the reference genome, to what extent this will affect the results of this study? For example, authors claimed "the non-reference TE insertions we detected are homogeneously distributed along chromosomes with no obvious pericentromeric bias (Fig. 1B)" (line 76-77). However, this observation could be due to the two possible causes: 1) reference bias, because of the difficulty to align two different genomes at the pericentromeric region, therefore, authors did not find the enriched TEs at the pericentromeric region in A. arenosa; 2) the comparison should include both reference-TEs and non-reference-TEs present in A. arenosa, instead of only non-reference-TEs.

2) Due to the same reason, the interpretation of other related analyses need to be updated as well, for example, Fig. 1C-H.

3) Lines 116 to 118, it is insufficient to claim "insertions located within these two particular genic compartments may more frequently be under positive selection rather than purifying selection". This pattern could be explained by purifying selection in another way, TE at 5' and 3' UTRs are functionally important and conserved, and deletion of TE will be deleterious. Here authors need to rephrase and explain more in detail somehow.

4) One important issue I could not follow, for autopolyploid, there are two sub-genomes, how authors interpret the TEs presence or absence. Although both sub-genomes are highly similar to each other, but some of TEs could be heterozygous, one sub-genome has TE insertion, another sub-genome not.

5) There are two major results in this study, one is the characteristics of TEs in either diploid or autopolyploid, another is the difference between diploid and autopolyploid. It is helpful to interpret your results in a big context, such as comparisons with studies in other closely related species and even well-studied drosophila.

6) Figure legends are not sufficient to follow, please explain more carefully, such as p values, which kinds of statistics authors used should be spelled out directly.

*We thank the reviewers for their very helpful comments and positive feedback. We have followed their unanimous recommendations by tempering some of our conclusions regarding adaptation, and by improving the readability and statistical clarity of our figure legends. We have also addressed each point raised as fully as possible, as detailed below.*

Responses to reviewers' comments:

Reviewer #1 (Remarks to the Author):

The manuscript submitted by Baduel et al. aims at deciphering the impact of autopolyploidy on TE-driven genome dynamics. The authors have exploited the genomic sequences of 300 Arabidopsis arenosa plants, among which 180 are polyploid.
As claimed by the authors, A. arenosa is a great system to study the short term genomme evolution of polyploidy because the origin of 4N A. arenosa is estimated to be ~ 60 ky. The comparative genomic approach between the diploid and tetraploid accessions is well done and its design is smart. The paper is well written, the science is sound and I only have minor critics to address to it :

1.1 - Throughout the manuscript, the authors are tempted to draw general conclusions from their results (a bad habit of arabidopsis genomicists indeed). I think they must at least temper their conclusions : a small genome is less prone to accumulate new insertions than a big one, simply because the probability that new insertions impede gene function is higher when the gene space is more prevalent....

*We tempered some of our conclusions (ll. 19-20, 189-190), in particular regarding adaptation (cf point 1.6). Of note, A. arenosa autotetraploids are still relatively recently derived (~60k years) so the gene space compared to the diploids is not radically different yet. However, given the accumulation of TEs we observe, we expect in the long term the intergenic space to be significantly increased, which could accelerate even further TE accumulation in the tetraploids. We now mention this prediction l. 219.*

1.2 - Even if the polyploidy is of recent origin, I think that it would be worthwhile to provide the reader with a phenetic tree of the the ~300 accessions used in this study. This can be very easily done using SNP data mined out from the raw seq.

*A neighbor-joining tree is available in Monnahan et al. 2019 and we now make specific reference to it in ll. 247-248.*

1.3 - authors have ignored in the introduction all the references on tobacco and rapeseed.

*We now mention the literature in tobacco and rapeseed (l. 208) as these are good examples of newly synthesized allotetraploids, in which the effect of polyploidy cannot be easily disentangled from that of hybridization.*

1.4 - p4 l100-102 : "further analysis revealed that the deficit of HF insertions within or near genes is most pronounced for copia, gypsy CACTA and hAT TE superfamilies which show an apparent insertion preference for genes especially exons...." please rephrase. This sentence is an oxymoron.

*We apologize for this confusion. In the revised manuscript we now specify more clearly (l.99) that the deficit of TE insertions within genes is observed at high-frequency and that the insertion preference for genes is measured at low-frequency.*

1.5 - p7 l167 : in polyplids : "further analysis of these local HF type A insertions indicated that they are specifically enriched for Copia LTR." the authors bring forward the propensity of Copia elements to insert into genes responsive to biotic stimuli. This is not the case for the diploids (see above). please clarify this.

*We now clarify l. 163 that the propensity of Copia elements to insert into genes is evaluated at low-frequency (i.e. before the insertion landscape is shaped too much by natural selection). This insertion preference for genes responsive to biotic stimuli is well observed for diploids (at low-frequency, Fig. S4) but indeed disappears at high-frequency. We attribute this difference to local positive selection for this category of Copia insertions specifically in tetraploids (l. 223).*

1.6 - Adaptation : the part on adaptation suffers over-interpretation. Association does not mean that there is a causal relationship between insertion and adaptation. The first step should be to conduct basic functional studies on this. Anyway, the link between early flowering and railway colonization is not obvious to a non specialist.

*Further work is indeed required to prove definitely a causal relationship, and we added a note ll. 189-190 to highlight this fact. However, a functional validation is beyond the scope of the present study. We also added a picture of a railway tetraploid (Fig. 5a), a plot illustrating the stark difference of flowering time between mountain and railway tetraploids (Fig. 5b), and a plot of FLC expression in mountain, mainland railway and BGS tetraploids (Fig. 5c) to illustrate more fully the strong association between railway colonization, early flowering, and loss of FLC expression (apart for BGS, see Baduel et al. 2018 for further details). This association is expected to enable better survival in the harsher environments provided by railways, which we now mention l. 172.*

conclusion : this paper should be published in nature communications. I could share the enthousiasm of the authors for this study, but certainly could also encourage them to rephrase some of their conclusions.

Reviewer #2 (Remarks to the Author):

Review of “Transposable elements over-accumulation in autopolyploids results from relaxed purifying selection and provides variants for rapid local adaptation”

This work by Baduel et al uses a published dataset of short Illumina sequences among diploids and autotetraploids (thereafter polyploids) of Arabidopsis arenosa to characterize copies of transposable elements (TEs; most main types). As in prior work on this system, the authors here circumvent the necessity of comparing diploids to experimental and natural polyploids by relying on range-wide populations. Contrasting with prior work focused on SNP, emphasis on TEs here yields original insights on possible causes and consequences of genome dynamics in polyploids. Given that limited work concluded on the extent at which whole-genome duplication (WGD) triggers or enables TE dynamics, such a study is of great interest and I read it with interest.
The manuscript is concise and well-written, although the presentation lacks of details to properly evaluate the significance of reported patterns. More comprehensive supporting

information should generally be provided (see comments below). Further justification of patterns involving the polyploid dataset would enable proper interpretation in the light of comparative genomics as well as population genetics. With a slightly increased structure helping non-specialists to follow the line of thought, the study is likely to offer convincing insights on interactions between TEs and WGD in shaping genome evolution.

Main comments

2.1 - L.49: A. arenosa should better introduced as to justify the assumption that the system enables to investigate "WGD independently of the confounding effects of hybridization". Given prior insights that the species hybridizes with Arabidopsis lyrata (Arnold et al. 2016 PNAS), I was here confused.

*Indeed, there are traces of hybridization with A. lyrata for some A. arenosa tetraploid populations, but this is comparatively low levels (only some populations have evidence of hybridization and in those where there is hybridization evident, it is at very low levels). There are populations with more (e.g. the one in the paper referenced, as well as ones in a newly reported study of a lyrate-arenosa hybrid zone), but these are not included here. The point we were trying to make is that contrary to allopolyploids that are the result of a hybridization event and thus 50% of the genome comes from a different species, A. arenosa tetraploids have either no, or only very low levels of hybridity, and thus we do not expect them to lead to ploidy-wide patterns.*

2.2 - The characterization of "non-reference TE insertions" must be further described and validated. First, the use of the outgroup A. lyrata to identify polymorphic TE copies in A. arenosa should be further justified. It is here based on an unpublished TE annotation of the A. lyrata genome (a summary would be welcome) and, at least, how TE copies were grouped into families should be described. The here presented dataset otherwise remains loosely defined and possible biases difficult to assess.

*As our pipeline can only detect TE sequences that are not present in the reference genome, which we refer to as non-reference TE insertions (see point 2.6 for deletions in A. lyrata), the use of the outgroup A. lyrata genome reduces considerably any bias for one ploidy vs another. Indeed, if a TE annotation were available from a diploid A. arenosa genome, our accuracy in detecting polymorphic TE copies in diploids would be greatly reduced as most TE insertions would have been already included in the annotation (and our pipeline does not detect absence variants). The TE annotation of the A. lyrata genome was completed by Legrand et al. and its description as well as their methods are now published in Legrand et al. Mobile DNA. "Differential retention of transposable element-derived sequences in outcrossing Arabidopsis genomes". 07/2019*

2.3 - Related to prior comment, provided specificities of TE calling, how individual sequencing coverage impacts on the accuracy of TE detection should be shown.

*Individual sequencing coverage is indeed the primary factor impacting the counts of non-reference TE insertions detected in any individual genome. That is why we included haplo-coverage, i.e. sequencing coverage by haploid genome, in our multiple linear model of non-reference TE content (Fig. 2f-3b).*

How does it affect the call of presence vs absence in diploids as well as polyploids?

*Our pipeline only calls presence variants compared to the reference genome, but little absence variation is expected within A. arenosa given the use of the outgroup A. lyrata genome as a reference (see point 2.2).*

How were heterozygotes treated in diploids vs polyploids (if not, this is to be stated transparently)?

*Given the high level of heterozygosity resulting from the obligate outcrossing of A. arenosa, it is likely that most non-reference TE insertions we detected are heterozygous and not homozygous. However, we could not distinguish between the two, and we added a statement to mention this limit ll.261-262.*

How was non-TE variation (e.g. indels) affecting the identification of homologous “TE insertions” taken into consideration in diploid vs polyploid individuals? Such clarification looks necessary to follow analyses and their interpretations.

*As A. arenosa polyploids are young (Arnold et al. 2016) in regard to the divergence between A. arenosa and A. lyrata, most non-TE variation compared to the reference A. lyrata genome is likely shared between diploids and polyploids and thus not affecting the identification of homologous TE insertions in a ploidy-specific manner.*

2.4 - The possible accumulation of non-reference TE copies along chromosome arms should be further justified in the light of possible biases against their detection across TE-rich chromosome regions. More specifically, how were pericentromeres here defined in A. arenosa should be briefly justified.

*Due to the lack of an available description of chromatin marks in A. lyrata, pericentromeric regions in A. arenosa were defined as regions where the density of reference TEs is higher than that of reference genes which corresponded to around 5Mbp around the centromeres (Fig. 1c). In these regions, we indeed expect detection of non-reference TE insertions to drop in sensitivity (which we now mention explicitly l. 82). Yet, at high-frequency non-reference TE insertions are enriched within pericentromeric regions (Fig. 1d). This demonstrates that the accumulation of non-reference TE copies within chromosome arms cannot be explained by detection biases alone as these should also affect high-frequency insertions.*

Confirmatory comparisons of gene-rich vs gene-poor regions of chromosome arms may strengthen assessment of purifying selection on TE copies.

*Following this suggestion, we counted LF and HF non-reference TE insertions within gene-poor (lower decile of density of reference genes) and gene-rich (upper decile of density of reference genes) 100kb regions in chromosome arms only. We confirmed the signal of purifying selection was not driven by the TE-rich pericentromeric regions as the proportion of non-reference TE insertions detected in gene-rich regions compared to gene-poor regions was significantly reduced at HF (50.2%) compared to LF (74.4%) (chi-2 p-value<1E-7).*

| | 2x-LF | 2x-HF |
|---|---|---|
| gene-poor | 70 | 109 |
| gene-rich | 204 | 110 |

*This information is now available ll.88-90 and as a supplementary table (table S1).*

2.5 - How were low-frequency (LF) were high-frequency (HF) TE copies defined should be

better described. Although the presented range-wide survey is reassuring, further justification of LF copies being recently inserted copies looks necessary (maybe on a subset of loci).

*The definition of LF and HF was calculated as the 1st and last decile of the diploid frequency spectrum (as indicated in Methods). We have included the frequency spectrum in both diploids and tetraploids with LF and HF insertions highlighted in Fig. S3a to clarify this definition. Unfortunately, with short-read technology we do not have access to the sequence of non-reference insertions apart from ~100bp on each side, which is not sufficient to estimate the age of the sequences based on SNPs.*

Note that Fig H-S2 and Fig F-H-S2 mentioned here were not accessible (not uploaded).

*The typos in ll. 103 & 105 were corrected to read Fig. 1h-S1c and Fig. 1f-h-S1c respectively.*

In particular, L. l87 states that HF and LF are parameters of age when selection is not a factor, but L93 says that selection is a factor. Please, clarify.

*We corrected l. 87 (now l. 84), as frequency can be used as a proxy for age when selection is strong and mostly purifying, which is the case here. Under this scenario, it is very rare for young insertions to be driven to high-frequency by positive selection and it is also unlikely for ancient insertions to be maintained at low frequencies by diversifying selection.*

2.6 - Not only the use of an outgroup reference genome (comments 2 and 3), but also the focus on natural populations makes it inherently difficult to properly distinguish copies out of de novo insertion from segregating copies. Accordingly, I would recommend to avoid wording such as “insertion” (e.g. “insertion preference”; L. 102) as it creates confusion. For instance, “insertion sites” in A. arenosa (L. 256) could in fact be deletions in A. lyrata (used as reference). As a whole, loci under scrutiny should be carefully described to support clarified presentation also for non-specialists.

*The scenario of a deletion in A. lyrata is indeed possible but the excision would need to be perfect for the presence variant to be detected as a non-reference insertion, yet this is rarely the case. In most of these situations, leftovers from the deletion in the reference genome would prevent the detection of the presence allele as a non-reference insertion.*

2.7 - The distinction of ‘type A’ vs ‘type B’ superfamilies should be thoroughly presented. ‘Type A’ (Copia, Gypsy, hAT or CACTA) and ‘type B’ (LINE, Mariner, MuDR or Harbinger) include TEs using different transposition mechanisms, having different sizes, aso… Their possible unification must therefore be further justified. Why is hAT classified as ‘type A’? Furthermore, as class I vs class II TEs may be expected to yield different patterns of “non-reference” copies (and transposons indeed show lots of non-reference copies here), such a clustering is surprising and should be further discussed; possibly regarding their timing of burst? Fig. 1h may suggest that type A proportions per category are similar to the whole genome proportions, contradicting the conclusion of “insertion preference for genes”. Comparison of the observed number of non-reference TEs per category to their expectation based on the fraction of the genome occupied by each category could
be provided for non-neutral insertion preference. Provided that the study relies heavily on the comparison of patterns between these ‘types’, such validation is crucial to follow corresponding results and offer a fully convincing paper.

*Type A and B superfamilies have been introduced to group together TE superfamilies that responded similarly to purifying selection, and thus also to the change of ploidy, despite their inherent differences in transposition mechanisms, sizes, etc. Thus, hAT was also classified*

*as type A and not as type B because hAT exonic insertions are significantly purged at high-frequency, following the same trend as Copia and Gypsy exonic insertions and even though the frequency of exonic insertions is much lower for hAT than for Copia or Gypsy (Fig. S2). Conversely, exonic insertions from type B superfamilies increased in proportion at high-frequency (LINE, Mariner, Harbinger) or were not affected (MuDR, Fig. S1c). In order to improve the transparency of this classification without impairing the readability of the manuscript we have added for each type A&B plot a supplementary figure with the corresponding plot with all superfamilies taken individually (Fig. S1c-S2-S3a).*

2.8 - Provided the use of an outgroup reference genome, the not-unexpected observation that TE copies "within or near (<250bp) genes but not further away" have an impact on their expressed should be further discussed. To what extent do issues related to micro-colinearity here affect such quantitative assessment? At least, this should be discussed in the light of prior survey in other Arabidopsis species that have reported longer-range effects (e.g. Holister et al. 2011. PNAS; Quadrana et al. 2016. Elife)

*The transcriptomic analysis is based on a previously generated RNAseq dataset where we can only infer carrier populations based on the WG sequencing data of independent individuals. Thus, based on this limitation, we have not been able to assess the more subtle long-range effects that were detected in A. thaliana.*

2.9 - When it comes to the comparison of diploids and polyploids (L.120ff), the study would greatly benefit from a more detailed description of proxies used in polyploids as well as their subsequent comparison to diploids. How sequencing coverage affected the detection of copies and dosage, and how LF vs HF were assessed at the population level should be spelled out to demonstrate the absence of detection bias in polyploids.

*TE content by individual is highly correlated with sequencing coverage (see point 2.3) and given that coverage by haploid genome is half in tetraploids what it is in diploids for the same library size we took it into account as a major effect predictor in our MLMs (Fig. 2f).*

*As the two frequency spectra did not significantly differ between diploids and tetraploids, we used the LF and HF thresholds defined in diploids to define LF and HF insertions in tetraploids as well. We now clarify this point ll. 271-274 and we added as a supplementary figure the comparison of the frequency spectrum by ploidy (Fig. S3a).*

2.10 - In my understanding, non-reference TEs were independently called in diploids and polyploids, and thus include a large amount of shared copies among ploidy in both datasets. To what extent such data fit assumptions of subsequently used parametric tests should be further justified (e.g. L. 123, L. 139, L. 147). Statistics used to test patterns shown in different figures is not always crystal clear and, in particular, the legend of Fig.2 should be clear regarding chi-square vs t-tests.

*We clarified in all the figure legends the statistics used in order to prevent any such confusion between statistical tests.*

The correspondence between text and Fig. 2G and between Fig. 2B and Fig. 2G is obscure. Terms like parm, pexon, putrs should be properly defined in the text.

*$p_{arm}$ is introduced in the text l.87, and we added definitions of $p_{exon}$ and $p_{UTRs}$ l.117 and l.120 respectively.*

More generally, as currently presented, effects seem rather low despite significant p-values. Provided that detection biases in polyploids can be ruled out, patterns tested (through e.g. resampling) among diploid-specific vs polyploid-specific copies may offer convincing insights.

*Indeed, a number of non-reference TE insertions we detect are shared between ploidies. Yet, we did not exclude them from comparisons between ploidies as this would generate an age-dependent bias in the comparison between diploids and tetraploids: tetraploid-specific insertions are necessarily younger than the tetraploids themselves while diploid-specific insertions can be as old as the divergence of A. arenosa from A. lyrata yet not sampled in the diploids.*

2.11 - Related to prior comment, Fig. 3c indicates that families (undefined; also see comment 2) show generally lower proportions of non-genic TE copies in polyploids as compared to diploids. In particular, it looks at odds with 35% of HF copies that are polyploid-specific (L. 147). This pattern remains confusing and possible underlying processes should be transparently discussed in the light of transposition vs frequency shifts (of LF vs HF) across ploidy levels.

*Indeed, polyploids have generally lower counts (Fig. 3a) and proportions (Fig. 3b) of non-genic HF TE copies than diploids, which is at odds with the scenario of a transposition burst in the neo-polyploid ancestor. However, this is not antagonistic with the 35% of HF TE copies being polyploid-specific as this is significantly less than in diploids (Fig. S3) and thus matches expectations from the genetic bottleneck associated with the WGD event.*

2.12 - Note that resampling of 100 accessions out of 105 diploid samples may be suboptimal to offer credible intervals. It should at least be justified.

*This minimum number of 100 individuals by resampling was necessary to obtain the 1% resolution in frequency required to differentiate LF insertions (LF-threshold=1.2%). We added this justification in the text ll.272-273.*

2.13 - Regarding the accumulation of Copia-related variants associated with local adaptation in polyploids, once issues of comment 9 have been clarified, population genetic structure should be further described. In particular, that enrichment of HF copies near genes of specific categories is not due to longer size of corresponding genes should be demonstrated to strengthen non-neutral claims.

*Immune response genes and in particular genes associated with response to stimulus (GO:0050896) are indeed longer than overall annotated genes. Yet, we found that genes harboring high-frequency clade-specific Copia insertions in tetraploids are not significantly longer than genes harboring any clade-specific Copia insertions in tetraploids, and this was true for all genes as well as for stimulus response genes. Following the reviewer's suggestion, we thus added a mention l. 167 to Fig. S4c confirming that enrichment of HF copies is not due to longer sizes of the corresponding genes.*

The mention of LF1, HF2 and HF3 across Fig 4 is confusing and should be further clarified.

*We clarified the confusing LF1, HF2, and HF3 denominations of clade-specific insertions used in Fig. 4 into private (1 carrier), shared (≥2 carriers), and high-frequency (≥3 carriers) respectively.*

2.14 - From Fig. 4D, it may be thought that Gypsy not Copia are showing most significant patterns in polyploids or hAT in diploids, suggesting that the focus here on Copia must be further justified.

*In Fig. 4d, only for Copia and in tetraploids did we detect any significant GO enrichment (FDR<0.05), while there were no significant patterns for Gypsy in polyploids nor for hAT in diploids.*

2.15 - The detailed characterization of the FLC region is highly complementary, while interesting in itself. How conclusive insights were reached from short read data should be further justified in the light of (L. 182) "local syntenic divergence between A. arenoas and A. lyrata" at this locus. The legend of Fig 5B is missing several abbreviations

*In order to circumvent the issues of local syntenic divergences at this locus, we remapped paired-end reads to an updated A. arenosa BAC sequence of the FLC region (see Material and Methods ll. 322-324). We added to the legend of Fig. 5 the missing abbreviations of the A. arenosa clades and of the carriers, non-carriers, and not-assigned.*

Reviewer #3 (Remarks to the Author):

This manuscript studied the TE dynamics following WGD based on ~300 individual Arabidopsis arenosa plants, including both diploids and autopolyploids. They identified 43,176 polymorphic TE insertions, and demonstrated that relaxed purifying selection rather than transposition bursts is the main driver of TE over-accumulation after WGD. In addition, TE insertion are enriched within or near abiotic and biotic stress response genes. Lastly, they showed TE insertion could affect a major flowering-time repressor gene and the mutation are correlated with the colonization of mainland railways. In general, this is a very interesting study about TE dynamics in diploid versus autopolyploid.

My comments:

3.1 - With A. lyrata as the reference genome, to what extent this will affect the results of this study? For example, authors claimed "the non-reference TE insertions we detected are homogeneously distributed along chromosomes with no obvious pericentromeric bias (Fig. 1B)" (line 76-77). However, this observation could be due to the two possible causes: 1) reference bias, because of the difficulty to align two different genomes at the pericentromeric region, therefore, authors did not find the enriched TEs at the pericentromeric region in A. arenosa; 2) the comparison should include both reference-TEs and non-reference-TEs present in A. arenosa, instead of only non-reference-TEs.

*Indeed, there is most likely a reference bias against detection of non-reference insertions within pericentromeric regions, which we mention explicitly l. 82. Yet, we could confirm that this was not driving the differences of distribution between LF and HF insertions (see point*

*2.4) and thus this was not preventing us from measuring the impact of purifying selection, which is the main focus of this manuscript.*

*We do not include reference TEs as our pipeline does not detect absence variants, and therefore we cannot differentiate within reference TEs those that are lyrata-specific from those that are ancestral to arenosa and lyrata. However, such ancestral TEs are very likely to be fossil and thus including them would not be informative regarding differential TE dynamics in diploids and tetraploids.*

3.2 - Due to the same reason, the interpretation of other related analyses need to be updated as well, for example, Fig. 1C-H.

*Regarding the first point, we have now added a supplementary analysis excluding pericentromeric regions (Table S1) to address the potential detection bias that can affect our analysis in these regions. Regarding the second point, we do not believe including reference TEs is justified in the light of our discussion in 3.1.*

3.3 - Lines 116 to 118, it is insufficient to claim "insertions located within these two particular genic compartments may more frequently be under positive selection rather than purifying selection". This pattern could be explained by purifying selection in another way, TE at 5' and 3' UTRs are functionally important and conserved, and deletion of TE will be deleterious. Here authors need to rephrase and explain more in detail somehow.

*Unlike A. thaliana, A. arenosa is an outcrosser with high levels of heterozygosity (Monnahan et al. 2019). Thus, most purifying selection against TE containing alleles will not require the deletion of the TE-sequences (which indeed could be deleterious if in functionally important regions like the UTRs) but by simply segregating out the allele altogether.*

3.4 - One important issue I could not follow, for autopolyploid, there are two sub-genomes, how authors interpret the TEs presence or absence. Although both sub-genomes are highly similar to each other, but some of TEs could be heterozygous, one sub-genome has TE insertion, another sub-genome not.

*A. arenosa is a pure autopolyploid with polysomic inheritance (Arnold et al. 2016), therefore there are no subgenomes per se as the four copies are completely equivalent. Nonetheless, there is most likely a high level of heterozygosity among the TE containing alleles that we detected. However, we were not able to distinguish between homozygous TE alleles and the different levels of heterozygous (see 2.3) and we now mention this limitation ll. 261-262.*

3.5 - There are two major results in this study, one is the characteristics of TEs in either diploid or autopolyploid, another is the difference between diploid and autopolyploid. It is helpful to interpret your results in a big context, such as comparisons with studies in other closely related species and even well-studied drosophila.

*We have added ll. 233-234 the missing references to the adaptive contributions of TEs that were elucidated in A. thaliana and Drosophila.*

3.6 - Figure legends are not sufficient to follow, please explain more carefully, such as p values, which kinds of statistics authors used should be spelled out directly.

*We have now clarified in all the legends the statistical tests used to generate the p-values mentioned in the figures.*

Reviewers' comments:

Reviewer #1 states in Remark to Editor section that (s)he is satisfied with the revision.

Reviewer #2 (Remarks to the Author):

This is the revised version of a manuscript that I recently reviewed for Nature Communication. I acknowledge that the authors have improved the presentation with an additional photo as well as clarifications regarding their analyses. The paper reads well and, although crucial information has to be extracted from supplementary analyses, is of interest for a generalist as well as a specialist audience. Such a survey of TE variation among related individuals is original and stimulating, making it a worth publication.
The authors offer detailed answers to several raised issues and, although they were unfortunately not always relayed in the core text, convincingly support their mains claims. In particular, clarification that boundaries of pericentromeres are unknown in A. arenosa leads to the presentation of new analyses comparing gene-rich vs gene-poor regions of chromosome arms that make the surmised impact of purifying selection on TE ‘insertions’ convincing. This is of great interest.
I further acknowledge that prior concerns regarding the loosely defined TE annotation used to call polymorphic TE copies in A. arenosa have been adequately addressed with a new citation (Legrand et al) and further justification for the use of a reference genome from the relative A. lyrata to possibly reduce biases in diploids vs polyploids. Such a survey of TE ‘insertions’ segregating across the distribution range of another species than A. thaliana is in itself of considerable originality.
The authors may be willing to elaborate on issues regarding the comparison of diploids vs polyploids before publication. The revised presentation of the approach to call TEs in diploids and polyploids now makes it clear that incomplete (or ‘dominant’) genotypes were here estimated (l.267). Provided the loss of information that inevitably increases with the ploidy level, I would have found it useful that subsequent comparisons based on frequencies among populations of diploids vs polyploids are also justified in the core text. In that context, a brief comment on the impact of 0.8 - 4X sequence coverage to assess loci in polyploids would be welcome.
Despite the similar frequency spectra of diploids and polyploids presented in supplementary analyses, comparisons of LF vs HF loci and how shared TE ‘insertions’ among ploidy were accounted remain elusive. I have not been able to grasp the strength of significant effects described in Figures 2 and 3 and to what extent chi-square and t-tests support biologically-meaningful conclusions. In any case, the line of thought and underlying assumptions would benefit from further description from patterns to conclusions. Accordingly, the authors may be willing to briefly comment on the significance of their findings and make sure that non-specialist readers are not confused.

Reviewer #3 (Remarks to the Author):

The revised manuscript largely addressed and explained all my previous concerns, and the manuscript is improved a lot.

[Editor: this reviewer provides more suggestions in Remark to Editor section, which were summarized below.]

(S)he thinks the revised manuscript still a few problems remain to be addressed:

1) The manuscript is still difficult to follow even I known TEs very well, this has been also pointed out by another reviewer in the first round;

2) Because of unable to infer the reference TE deletions, another half of polymorphic TEs, at least some of the conclusions of this study will be affected to some extent.

3) Authors treated homologous TE allele and heterozyous allele as the same, this will be misleading on the analyses of population genetics, which largely relies on the frequency of alleles (here TE insertions). Especially, the frequency related parameters HF and LF between diploid and tetraploid are crucial to infer the main conclusions, the situation might be even worse.

Reviewer #4 (Remarks to the Author):

This article presents the study of resequencing data from ~300 individuals of Arabidopsis arenosa to explore driving forces that affect TEs distribution after whole genome duplication (WGD). The authors show that relaxed purifying selection rather than transposition burst are responsible of TE accumulation after WGD. Some of these new variants likely contribute to local adaptation. I found the paper convincing, although difficult to read. The following recommendation might help the reader.

The author should provide some basic knowledge of TE population dynamics between autotetraploid and diploid populations. For example, TE population dynamics, as any mutation population dynamics, is expected to be slower in autotetraploids compared to diploids. In addition, expected reduced purifying selection in autotetraploids and increased TE copies (doubling of genome content) is expected to allow for both higher numbers and higher frequencies of TE insertions. Consequently, some argument should be presented to state that tetraploid and diploid A. arenosa lineages can be compared. I found figure 2c showing similar trends for parms helpful to ague for similar frequency spectrum in other compartments than exons, indicating that they reach probably a similar state.

The methodology relies on the identification of TEs where homozygous or heterozygous insertion cannot be distinguished. Consequently, the term of allele frequency used sometimes by the author is often misleading (in particular page 11, line 276). My understanding is that they calculate a proportion of individuals that contain a TE insertion in a given locus. Consequently, I suggest to replace this term by something such as individual-level TE insertion frequency.

Page 4, line 97 state that they found a transcriptomic impact of TE insertions on gene expression. The term of "impact" should be change by "interaction" as it cannot be ruled out by their results that TEs insert preferentially near genes with low expression level.

Page 5, line 116 state that UTRs tend to be under positive selection. However, it cannot be ruled out that it exist an insertional bias. This alternative hypothesis should be mentioned.

Hadi Quesneville

*We would like to thank again the reviewers for their very insightful comments and positive feedback. Following a shared concern about the importance of zygosity, we now explicitly state that our study concerns carrier, not allele frequencies and explain in greater detail why this is so. We have also followed the reviewers' recommendations to further develop the description of our results to improve the readability. Below you will find our point by point response to the reviewers' comments.*

Reviewers' comments:

Reviewer #1 states in Remark to Editor section that (s)he is satisfied with the revision.

Reviewer #2 (Remarks to the Author):

This is the revised version of a manuscript that I recently reviewed for Nature Communication. I acknowledge that the authors have improved the presentation with an additional photo as well as clarifications regarding their analyses. The paper reads well and, although crucial information has to be extracted from supplementary analyses, is of interest for a generalist as well as a specialist audience. Such a survey of TE variation among related individuals is original and stimulating, making it a worth publication.

The authors offer detailed answers to several raised issues and, although they were unfortunately not always relayed in the core text, convincingly support their mains claims. In particular, clarification that boundaries of pericentromeres are unknown in A. arenosa leads to the presentation of new analyses comparing gene-rich vs gene-poor regions of chromosome arms that make the surmised impact of purifying selection on TE 'insertions' convincing. This is of great interest.

I further acknowledge that prior concerns regarding the loosely defined TE annotation used to call polymorphic TE copies in A. arenosa have been adequately addressed with a new citation (Legrand et al) and further justification for the use of a reference genome from the relative A. lyrata to possibly reduce biases in diploids vs polyploids. Such a survey of TE 'insertions' segregating across the distribution range of another species than A. thaliana is in itself of considerable originality.

2.1 - The authors may be willing to elaborate on issues regarding the comparison of diploids vs polyploids before publication. The revised presentation of the approach to call TEs in diploids and polyploids now makes it clear that incomplete (or 'dominant') genotypes were here estimated (l.267). Provided the loss of information that inevitably increases with the ploidy level, I would have found it useful that subsequent comparisons based on frequencies among populations of diploids vs polyploids are also justified in the core text. In that context, a brief comment on the impact of 0.8 - 4X sequence coverage to assess loci in polyploids would be welcome.

*We have further developed how the loss of sensitivity resulting from increased ploidy (half the coverage per haploid genome) is expected to affect our analysis and particularly our cross-ploidy comparisons. First, we have added a note ll. 323-327 about the loss of sensitivity directly associated with drops in haplo-coverage, and thus why we have added haplo-coverage as a parameter in our multiple linear models (MLMs) of TE content. Second, we have made explicit (ll. 295-309) the frequency-dependent biases induced by reduced*

*haplo-coverage in tetraploids and further justified our use of carrier-frequencies for cross-ploidy comparisons.*

2.2 - Despite the similar frequency spectra of diploids and polyploids presented in supplementary analyses, comparisons of LF vs HF loci and how shared TE 'insertions' among ploidy were accounted remain elusive. I have not been able to grasp the strength of significant effects described in Figures 2 and 3 and to what extent chi-square and t-tests support biologically-meaningful conclusions. In any case, the line of thought and underlying assumptions would benefit from further description from patterns to conclusions. Accordingly, the authors may be willing to briefly comment on the significance of their findings and make sure that non-specialist readers are not confused.

*Following a related suggestion from reviewer #4 (see point 4.1) we further developed our description and interpretation of the effects described in Figure 2. We now insist on the limited differences observed between ploidies outside of exons, which confirms that the TE landscapes remain globally similar in diploids and tetraploids (ll. 137-141). We now also describe in more detail the other results of Figure 2 and 3 (ll. 142-143, 149,156-160) to provide more context to the conclusions they enable us to draw. In the same vein, we have removed the left panel of Fig. 3a (LF TE content), as the focus of the Fig. 3 is on HF TE insertions, notably those that are non-genic. For this reason, Fig. 3b is now only showing results of the MLM obtained for the non-genic TE content. Together, these modifications clarify the strength of the effects observed and their biological significance.*

Reviewer #3 (Remarks to the Author):

The revised manuscript largely addressed and explained all my previous concerns, and the manuscript is improved a lot.

[Editor: this reviewer provides more suggestions in Remark to Editor section, which were summarized below.]

(S)he thinks the revised manuscript still a few problems remain to be addressed:

3.1 - The manuscript is still difficult to follow even I known TEs very well, this has been also pointed out by another reviewer in the first round;

*We have followed the reviewers' suggestions to improve the readability of the manuscript, in particular by developing further the theoretical expectations from population genetics of tetraploids in the introduction (ll. 34-37 & 45-50).*

3.2 - Because of unable to infer the reference TE deletions, another half of polymorphic TEs, at least some of the conclusions of this study will be affected to some extent.

*We are indeed unable to detect deletions of reference TE sequences. However, only a minor fraction of them is expected to be polymorphic among A. arenosa individuals given that the reference genome we used is that of the outgroup A. lyrata. In other words, most reference TEs should be fixed or else fully absent in A. arenosa.*

3.3 - Authors treated homologous TE allele and heterozyous allele as the same, this will be misleading on the analyses of population genetics, which largely relies on the frequency of alleles (here TE insertions). Especially, the frequency related parameters HF and LF

between diploid and tetraploid are crucial to infer the main conclusions, the situation might be even worse.

*Following a related suggestion of Reviewer #4 (see point 4.2), we have clarified (ll.92-94 & 286-287) that our methodology is limited to the estimation of carrier, not allele frequencies. As suggested in point 2.1, we now provide a more detail description of the biases that affect the estimation of allelic frequencies in tetraploids and we further justify our use of carrier-frequencies for cross-ploidy comparisons (ll. 295-309).*

Reviewer #4 (Remarks to the Author):

This article presents the study of resequencing data from ~300 individuals of Arabidopsis arenosa to explore driving forces that affect TEs distribution after whole genome duplication (WGD). The authors show that relaxed purifying selection rather than transposition burst are responsible of TE accumulation after WGD. Some of these new variants likely contribute to local adaptation. I found the paper convincing, although difficult to read. The following recommendation might help the reader.

4.1 - The author should provide some basic knowledge of TE population dynamics between autotetraploid and diploid populations. For example, TE population dynamics, as any mutation population dynamics, is expected to be slower in autotetraploids compared to diploids.

*We have added a paragraph in the introduction (ll. 45-50) to provide some theoretical expectations about the impacts of polyploidy on mutations dynamics, depending on their deleterious / beneficial effects or on their dominance and the consequences on the rates of adaptation.*

In addition, expected reduced purifying selection in autotetraploids and increased TE copies (doubling of genome content) is expected to allow for both higher numbers and higher frequencies of TE insertions.

*We now mention these expectations in the introduction ll. 34-38.*

Consequently, some argument should be presented to state that tetraploid and diploid A. arenosa lineages can be compared. I found figure 2c showing similar trends for parms helpful to ague for similar frequency spectrum in other compartments than exons, indicating that they reach probably a similar state.

*We have further highlighted in the introduction (ll.58-60) that tetraploid and diploid A. arenosa lineages can be compared thanks to the young age of the tetraploids and their largely overlapping ecological niche, and we have expanded our interpretation of Fig. 2 to include this important point (ll. 137-141).*

4.2 - The methodology relies on the identification of TEs where homozygous or heterozygous insertion cannot be distinguished. Consequently, the term of allele frequency used sometimes by the author is often misleading (in particular page 11, line 276). My understanding is that they calculate a proportion of individuals that contain a TE insertion in a given locus. Consequently, I suggest to replace this term by something such as individual-level TE insertion frequency.

*This is correct, and we have replaced the mention of allele frequency used l.294 (former l. 276) with carrier frequency as well as in the main text (l. 92). Following similar comments*

*(see points 2.1 and 3.3) we describe in more detail (ll. 295-309) the different effects that could influence the estimation of allele frequency from carrier frequency depending on ploidy.*

4.3 - Page 4, line 97 state that they found a transcriptomic impact of TE insertions on gene expression. The term of “impact” should be change by “interaction” as it cannot be ruled out by their results that TEs insert preferentially near genes with low expression level.

*We have changed our wording accordingly l. 103, yet we are comparing expression of carriers and non-carriers gene by gene and therefore the interaction cannot be due to preferential insertion within genes with low expression levels. However, we cannot rule out the lower expression of the TE-associated haplotype before the TE inserted as haplotyping tools are not yet available for tetraploid genotypic data. We now mention this alternate hypothesis ll. 105-107.*

4.4 - Page 5, line 116 state that UTRs tend to be under positive selection. However, it cannot be ruled out that it exist an insertional bias. This alternative hypothesis should be mentioned.

*Insertional preferences are observed with the least bias at low-frequency but here we observed an increased proportion of TE insertions within UTRs at high-frequency compared to low-frequency. Although, we cannot rule out alternative hypotheses, our results are most consistent with positive selection. We have nonetheless rephrased the corresponding sentence in the text to further tone down our conclusion l. 123.*

REVIEWERS' COMMENTS:

Reviewer #2 (Remarks to the Author):

I acknowledge that the authors now provide sufficient information to enable readers to appreciate here reached conclusions. With main challenges inherent to the genotyping of TE ‘insertions’ in tetraploids and biases due to the lack of zygosity estimates for alleles being spelled out (mostly, L. 295ff), non-specialist readers can now properly evaluate comparisons of diploids vs polyploids. Accordingly, the manuscript looks mature for publication together with the correspondence out of peer-review.

As the authors have a last opportunity to provide minor amendments, I would suggest to consider the following issues:
-Possible overestimation of the frequency of rare recessive alleles based on carrier frequency in polyploids should be briefly mentioned before corresponding results are presented (i.e. around L. 130) or, at least, when intermediate conclusions are reached (L. 168). Having to ‘wait for’ the presentation of methodological details to know about biases indeed raises late questions and may be suboptimal. I guess that it would be easier to follow the significance of results with such caveats in mind from the start.
-L.155: “when non-genic” is unclear; words may be missing.
-On L. 255, the authors mention “major over-accumulation of TEs […] in the autotetraploids”. I would suggest to be slightly more specific and to further justify “major”. In particular, a clear-cut conclusion on the impact of relaxed purifying selection follows and is appended with another statement that “resulting increase in genetic load remained subtle”. It may not be easy to distinguish major from subtle based on statistical effects at this stage and, maybe, some such claims should be discussed as perspectives.

I congratulate the authors on a nice study that appears consistent with a significant role of purifying selection in shaping TE distribution (as was recently shown in other Arabidopsis as well as Arabis species) and that supports predictions of limited TE burst in autopolyploids (I believe that this was initially anticipated in C Parisod & N Senerchia. 2012. Responses of transposable elements to polyploidy. In Plant Transposable Elements (eds M-A Grandbastien & J M Casacuberta, 147–168. Topics in Current Genetics. Berlin, Heidelberg: Springer Berlin Heidelberg).

Christian Parisod, with Rimjhim Roy Choudhury

Reviewer #3 (Remarks to the Author):

The authors have addressed all my concerns, and I am satisfied with the revised manuscript.

Reviewer #4 (Remarks to the Author):

The new manuscript addresses all the point I raised in a very satisfactory manner. It reads well and all results are much more convincing.

REVIEWERS' COMMENTS:

Reviewer #2 (Remarks to the Author):

I acknowledge that the authors now provide sufficient information to enable readers to appreciate here reached conclusions. With main challenges inherent to the genotyping of TE 'insertions' in tetraploids and biases due to the lack of zygosity estimates for alleles being spelled out (mostly, L. 295ff), non-specialist readers can now properly evaluate comparisons of diploids vs polyploids. Accordingly, the manuscript looks mature for publication together with the correspondence out of peer-review.

As the authors have a last opportunity to provide minor amendments, I would suggest to consider the following issues:
-Possible overestimation of the frequency of rare recessive alleles based on carrier frequency in polyploids should be briefly mentioned before corresponding results are presented (i.e. around L. 130) or, at least, when intermediate conclusions are reached (L. 168). Having to 'wait for' the presentation of methodological details to know about biases indeed raises late questions and may be suboptimal. I guess that it would be easier to follow the significance of results with such caveats in mind from the start.

*We added a note l. 144 to briefly mention the potential biases that could affect differently the detection of TE insertions in autotetraploids but that nonetheless the TE landscapes captured in diploids and tetraploids remain globally similar.*

-L.155: "when non-genic" is unclear; words may be missing.

*We have re-read that sentence and found no word missing.*

-On L. 255, the authors mention "major over-accumulation of TEs […] in the autotetraploids". I would suggest to be slightly more specific and to further justify "major". In particular, a clear-cut conclusion on the impact of relaxed purifying selection follows and is appended with another statement that "resulting increase in genetic load remained subtle". It may not be easy to distinguish major from subtle based on statistical effects at this stage and, maybe, some such claims should be discussed as perspectives.

*We now clarify ll. 242-243 that the subtle increase in genetic load is the conclusion of our previous study (reference 22) where only SNPs were taken into account. On the opposite, in the present study the increase in genetic load at the level of TE-insertions is on another order of magnitude, hence our use of the word major to describe the "over-accumulation of TEs specifically within or near genes" (l. 233).*

I congratulate the authors on a nice study that appears consistent with a significant role of purifying selection in shaping TE distribution (as was recently shown in other Arabidopsis as well as Arabis species) and that supports predictions of limited TE burst in autopolyploids (I believe that this was initially anticipated in C Parisod & N Senerchia. 2012. Responses of transposable elements to polyploidy. In Plant Transposable Elements (eds M-A Grandbastien & J M Casacuberta, 147–168. Topics in Current Genetics. Berlin, Heidelberg: Springer Berlin Heidelberg).

Christian Parisod, with Rimjhim Roy Choudhury

*We thank Christian Parisod and Rimjhim Roy Choudhury for their comments and their appreciation of our work. We also thank them for pointing to us the above reference, which we now cite.*

Reviewer #3 (Remarks to the Author):

The authors have addressed all my concerns, and I am satisfied with the revised manuscript.

Reviewer #4 (Remarks to the Author):

The new manuscript addresses all the point I raised in a very satisfactory manner. It reads well and all results are much more convincing.
